# Supplementary material for: Rapid self-heating synthesis of Fe-based nanomaterial catalyst for advanced oxidation
Source: Nat Commun. 2023 Aug 17;14:4975. doi: 10.1038/s41467-023-40691-2 (PMC10435566; doi:10.1038/s41467-023-40691-2)
Supplement: Supplementary file 1 — Supplementary Information [file 41467_2023_40691_MOESM1_ESM.pdf]

Supplementary Information for

Rapid self-heating synthesis of Fe-based nanomaterial catalyst for advanced oxidation

Fengbo Yu[1], Chao Jia[1], Xuan Wu[1], Liming Sun[1], Zhijian Shi[1], Tao Teng[1], Litao Lin[1], Zhelin He[1], Jie Gao[1], Shicheng Zhang[1,2], Liang Wang[3], Shaobin Wang[4,*] and Xiangdong Zhu[1,5,*]

[1]Shanghai Technical Service Platform for Pollution Control and Resource Utilization of Organic Wastes, Shanghai Key Laboratory of Atmospheric Particle Pollution and Prevention (LAP3), Department of Environmental Science and Engineering, Fudan University, Shanghai 200092, China.

[2]Shanghai Institute of Pollution Control and Ecological Security, Shanghai 200092, China.

[3]School of Energy and Power, Jiangsu University of Science and Technology, Zhenjiang, 212003, China.

[4]School of Chemical Engineering and Advanced Materials, The University of Adelaide, Adelaide, SA 5005, Australia.

[5]National & Local Joint Engineering Laboratory for Municipal Sewage Resource Utilization Technology, Suzhou University of Science and Technology, Suzhou, 215009, China.

Contents:

Supplementary Materials and Methods

Supplementary Figures from 1 to 38

Supplementary Tables from 1 to 10

Supplementary References

Materials and Chemicals

Rice straw for preparing a soft carbon substrate was collected from Shandong province in China. Ferrous sulfide (FeS, Tech), ferrous sulfate ($FeSO_4 \cdot 7H_2O$, 99%), iron acetate ($C_4H_7FeO_5 \cdot nH_2O$, AR), iron carbide (Fe_3C, 99.5%), carbon black (super p li, super high density), sodium peroxydisulfate ($Na_2O_8S_2$, 99%), methanol (CH_4O, 99.8%), chloramphenicol (CAP, 98%), tert-butanol (TBA, 99%), potassium iodide (KI, 98.5%), hydrochloric acid (HCl, 36%), ammonium iron (II) sulfate ($(NH_4)_2FeSO_4 \cdot 6H_2O$, 99.5%), sulfuric acid (H_2SO_4, 95%), hydroxylamine hydrochloride ($NH_2OH \cdot HCl$, 98.5%), acetic acid ($C_2H_4O_2$, 99.5%), ammonium acetate (CH_3COONH_4, 98%), and sodium hydrate (NaOH, 96%) were purchased from Sinopharm Chemical Reagent Co., Ltd. Other chemicals, p-hydroxybenzoic acid ($C_7H_6O_3$, 99%), 1,10-phenanthroline monohydrate ($C_{12}H_8N_2 \cdot H_2O$, 99%), benzoic acid ($C_7H_6O_2$, 99.5%), and iron oxide (γ-Fe_2O_3, 99.5%) were obtained from Aladdin Industrial Corporation.

IR spectrometer and blackbody radiation fitting

During the FJH process, the black body radiation from the sample was collected by an optical fiber, and the spectral radiation was recorded by an IR spectrometer (FX2000, Ideaoptics, China) at 500-1100 nm. Before the measurement, the temperature was calibrated with a mercury lamp[1].

Analysis of lighting intensity

Lighting intensity was recorded by a high-speed camera in the FJH process. The lighting intensity of different reacted positions was first acquired based on the HSV color model by MATLAB software. Then, the change of lighting intensity with time and reacted position can be obtained.

Characterizations

The crystalline structures of Fe-based material were analyzed by X-ray diffraction (XRD, X'Pert PRO MPD) with a Cu Kα X-radiation. The element species of Fe-based material were measured by X-ray photoelectron spectroscopy (XPS, Thermo Kalpha), and the spectra were corrected by the C *1s* at 284.6 eV. Transmission electron microscopy (TEM, FEI Tecnai F20) and spherical correction transmission electron microscope (sc-TEM) were performed to analyze the dispersion of elements and particle size. ^{57}Fe Mössbauer spectra (Wissel, Germany) were recorded at room temperature by a proportional counter with a ^{57}Co (Pd) radioactive source. X-ray absorption spectra (XAS) including X-ray absorption near-edge structure (XANES) and extended X-ray absorption fine structure (EXAFS) of the Fe-based material at Co K-edge (7709 eV) were collected at the Singapore Synchrotron Light Source (SSLS) center, where a pair of channel-cut Si (111) crystals were used in the monochromator. The Fe K-edge XANES data was recorded in a transmission mode. Fe foil, FeS, FeO, and Fe_3O_4 were used as reference standards. The storage ring was working at the energy of 2.5 GeV with an average electron current of below 200 mA. The acquired EXAFS data was extracted and processed according to

the standard procedures using the ATHENA module implemented in the FEFIT software packages[2, 3]. The k^3-weighted Fourier transform (FT) of x (k) in R space was obtained over a range of 0-14.0 Å by applying a base window. Raman spectroscopy (LabRam HR Evolution) was performed to analyze the degree of graphitization. Nitrogen adsorption-desorption isotherms and surface areas were acquired by a BET analyzer (Quadrasorb SI, America). The elemental (C, H, N, S) compositions were measured by an elemental analyzer (Vario ELIII, Germany Elemental Instrument Co., Ltd). O element composition was acquired by the difference. The measurement of ash content was referred to literature[4].

Degradation of chloramphenicol

CAP degradation in aqueous solutions: Degradation of chloramphenicol was performed in a batch reactor. A Fe-based material (25 mg) and sodium peroxydisulfate (41.7 mg) were added to a centrifuge tube with 25 mL chloramphenicol solutions (60 ppm), and the initial pH is about 3. The Fe-based material or sodium peroxydisulfate was added independently into a centrifuge tube with 25 mL chloramphenicol solutions (60 ppm) as control. After that, the centrifuge tube was put in an oscillation box with 150 rpm at 28 ^{o}C. The solutions were taken at a specific time, filtered through a 0.22 μm filter, and added immediately with an equal volume of methanol to prevent a reaction before measurements. The concentrations of chloramphenicol were detected rapidly by High-performance liquid chromatography (HPLC) at a wavelength of 278 nm, 1 mL min^{-1} mobile

phase (the volume ratio of methanol to ultrapure water is 4:6), and the column temperature is 25 ^{o}C.

CAP degradation in the soil slurry: Degradation of chloramphenicol was performed in a batch reactor. A Fe-based material (25 mg), sodium peroxydisulfate (41.7 mg), and soil (2500 mg) were added to a centrifuge tube with 25 mL chloramphenicol solutions (60 ppm) without regulating pH. The soil and sodium peroxydisulfate were added into a centrifuge tube with 25 mL chloramphenicol solutions (60 ppm) without Fe-based material as control. After that, the degradation process is the same as CAP degradation in aqueous solutions. The degradation process of chloramphenicol in three types of soil (red soil, yellow soil, and black soil) were conducted.

Cyclic voltammetry and electrochemical impedance spectroscopy measurements

A Fe-based material coated glassy carbon electrode (using Nafion perfluorinated resin solution as binder) was selected as the working electrode. Ag/AgCl electrode and Pt electrode were used as reference and counter electrodes, respectively. A mixed solution of sodium peroxydisulfate (7 mM) and sodium sulfate (0.2 M) was used as an electrolyte. The cyclic voltammetry curve was recorded at the potential range from -1.1 to 0.2 V. The scan rate was 10 mV s^{-1}. Electrochemical impedance spectroscopy was also measured. Low frequency and high frequency were 0.01 and 100000, respectively.

Analysis of reactive intermediates

Detection of iron species: Total dissolved iron and Fe (II) concentrations in the degradation process were detected using the standard method (HJ/T 345-2007). Generally, filtered samples (1 mL), 1,10-phenanthroline (2 mL, 0.5 wt%), and a buffer solution of acetic acid and ammonium acetate (5 mL) were mixed. After that, the mixed solution was diluted to 50 mL with deionized water. Finally, Fe (II) concentrations were detected by a UV-vis spectrometer at 510 nm. For the total iron concentrations, a similar technique as above was employed except that hydroxylamine hydrochloride (1 mL, 0.5 g mL^{-1}) was also added to the mixed solution.

Electron paramagnetic resonance (EPR) analysis: 5,5-dimethyl-1-pyrroline N-oxide (DMPO) was used as the $SO_4^{\cdot -}$ and ·OH trapping reagent and 2,2,6,6-tetramethyl-4-piperidone (TEMP) was used as the $^1O_2$ trapping reagent[5]. Generally, Fe-based material (25 mg) and sodium peroxydisulfate (41.7 mg) were added to a centrifuge tube with 25 mL chloramphenicol solutions (60 ppm). After that, the centrifuge tube was put in an oscillation box with 150 rpm. The solutions were taken at 5 min, filtered through a 0.22 μm filter, and tested for radicals by an EPR spectrometer (Bruker EMXplus).

Radical quenching tests: Radical quenching experiments were also tested. Tert-butanol (TBA) was a strong quencher of ·OH, methanol was a strong quencher of ·OH and $SO_4^{\cdot -}$, and potassium iodide was a quencher of surface radical of Fe-based material.

Quantitative analysis of hydroxyl radical: the measurement was conducted using benzoic acid as a probe. After the reaction of ·OH with benzoic acid, p-hydroxybenzoic acid (p-

HBA) was produced and its concentration was measured by HPLC. The method and calculation of cumulative ·OH concentration were referred to literature[6] as equation (1).

$$\text{Cumulative } [\cdot OH] \text{ produced} = [p\text{-}HBA] \times 5.87 \quad (1)$$

Intermediate detection by GC-MS and HPLC-QTOF-MS

After the CAP degradation process of 15 min, the reacted solution (50 mL) was extracted with dichloromethane (5 mL). The intermediate products were detected by GC-MS. The detailed information can be referred to this literature[7]. HPLC-QTOF-MS with an ESI source in the negative ionization was used for analysis. An Agilent Eclipse C18 column (3.0 mm x 150 mm, 1.8 μm) was used for separation. An injection volume was 3.0 μL and acetonitrile with 0.1% formic acid was selected as the mobile phase at a flow rate of 3 mL min^{-1}. The m/z range was 50~1500. The capillary voltage of the ESI was set at 3500 V, the gas temperature was set to 325 ^{o}C, the drying gas flow was 12 L min^{-1}, and the nebulizer was 60 psi. The inorganic substances of chloride and nitrate were measured by ion chromatography (ICS-5000).

Gas production detection from the FJH process by GC-MS and HPLC

The CS_2 and S_2 gas from FJH processes were collected by ethanol and CS_2 solvent condensation according to the literature[8]. The CS_2 was detected by GC-MS (Agilent 5977B GC/MSD) with an HP-Wax column. The GC oven temperature was programmed at a rate of 30 ^{o}C min^{-1} from 60 ^{o}C (held for 2 min) to 230 ^{o}C (held for 5 min). The carrier gas was helium with a flow rate of 1 mL min^{-1}, and 1 μL sample was injected at 230 ^{o}C.

S_2 concentration was detected rapidly by HPLC at a wavelength of 254 nm, 0.8 mL min^{-1} mobile phase (the volume ratio of methanol to ultrapure water is 95:5), and the column temperature is 25 ^{o}C.

Density functional theory (DFT) calculation

Explanation of input structure. XRD and ^{57}Fe Mössbauer spectra results indicated the existence of FeS and Fe^0. STEM-EDS and STEM-EELS further proved the formation of the Fe^0/FeS heterostructure which was an important catalytic structure. The formation reason was that Fe^0 was produced from fast breaking Fe-S bond and partial Fe^0 was sharply solidified near FeS for forming Fe^0/FeS heterostructure due to the rapid cooling at a cooling rate. Meanwhile, the Fe^0/FeS heterostructure was embedded in thin-bedded graphene which was testified by the high value of I_{2D}/I_G from Raman, C *1s* XPS, and HRTEM.

In addition, the PDS activation mechanism of Fe-based materials generally involved PDS adsorption on metal sites and the subsequent reduction of PDS to produce reactive free radicals for CAP degradation. The Fe^0/FeS heterostructure embedded in thin-bedded graphene was the main contributor to CAP degradation. Therefore, the system of PDS adsorption on Fe^0/FeS heterostructure embedded in thin-bedded graphene (Fe^0/FeS/C or FeS/Fe^0/C) was structured for DFT analysis. To explain the superior activation performance of Fe^0/FeS heterostructure on PDS, we compared FeS embedded in thin-bedded graphene (FeS/C) and Fe^0 embedded in thin-bedded graphene (Fe^0/C). The role

of thin-bedded graphene was further explained by structuring the FeS (FeS without graphene) and FeS embedded in thin-bedded graphene (FeS/C) respectively.

Meanwhile, the reduction of PDS on the material to produce reactive free radicals was testified, which produced the Na_2SO_4 and ·OH due to the O-O bond breakage of $Na_2S_2O_8$. Therefore, the PDS activation reactions on Fe^0/C and Fe^0/FeS/C from the beginning to the end were constructed to evaluate the energy barrier for the O-O bond breakage and Gibbs free energy for the whole reaction process.

Calculation methods. DFT calculation was performed by a Vienna ab initio simulation package (VASP) using the projector augmented wave (PAW) method[9, 10, 11]. The exchange correlation potential was represented by the Perdew–Burke–Ernzerhof (PBE) functional within the generalized gradient approximation (GGA)[12]. DFT calculation evaluated the adsorption configuration of FeS, FeS/C, Fe^0/C, Fe^0/FeS/C, and FeS/Fe^0/C with PDS, FeS crystal with a (110) surface, Fe crystal with a (011) surface, and C representing graphene. 4 x 7 x 1 super cell of graphene was structured, and 3 x 5 x 1 super cell of Fe and FeS was structured for DFT calculation. To eliminate the interactivity of the adjoining slab model, the vacuum layer was set to 15 Å. The cutoff energy for the plane-wave-basis was set to be 500 eV. The k-point sampling grid was set to 3×3×1. The convergence tolerances of energy and force were set to 1.0×10^{-4} eV $atom^{-1}$ and 1.0×10^{-2} eV $Å^{-1}$, respectively. The DFT-D3 method was used to describe the van der Waals

(vdW) interactions between substrates and adsorbate[13]. The adsorption energy (E_{ad}) of $Na_2S_2O_8$ on heterostructure can be calculated by equation (2):

$$E_{ad} = E\ (sub+Na_2S_2O_8) - E\ (sub) - E\ (Na_2S_2O_8) \quad (2)$$

Where, E (sub+$Na_2S_2O_8$) and E (sub) are the total energy of the FeS (110)/C or Fe (011)/C heterostructure with and without $Na_2S_2O_8$, respectively, and E ($Na_2S_2O_8$) is the energy of $Na_2S_2O_8$.

The transition state of O-O bond breakage in the adsorption configuration of Fe^0/C and Fe^0/FeS/C with PDS was calculated according to the reacted equation as equation (3). The transition state method was CINEB.

$$Na_2S_2O_8{*}A + 2\ H_2O + 2Na^{2+} \longrightarrow 2\ Na_2SO_4{*}A + 2\ {\cdot}OH + 2\ H^+ \quad (3)$$

A represented the Fe-based material, Fe^0/C, and Fe^0/FeS/C. The Gibbs free energy of this reaction was also calculated according to equations (4) and (5).

$$\Delta G = 2\ G\ (NA_2SO_4{*}A) + 2\ G\ ({\cdot}OH) + G\ (H_2) - G\ (NA_2S_2O_8{*}A) - 2\ G\ (H_2O) - 2\ G\ (Na^{2+}) \quad (4)$$

$$G = E_{tot} + E_{zpe} + T\ \Delta S \quad (5)$$

E_{tot} represents the total energy of the optimized structure. T was 298.15 K, E_{zpe} represents the zero point vibration energy, and ΔS represents enthalpy change.

Automation equipment

For large-scale production of superior Fe-based material, the automation equipment was designed. The automation equipment includes a loading and collecting area, robot arm, and reacted technics area. After loading, the raw material was transferred into the reacted

area by the robot arm. Subsequently, a current was driven in the material by the triggered voltage, and a reaction was achieved to improve the material structure as a superior Fe-based material. Finally, the robot arm further transfers Fe-based material from the reacted technics area to the collecting area. After completion, the next steps are circulatory to continuously produce Fe-based material.

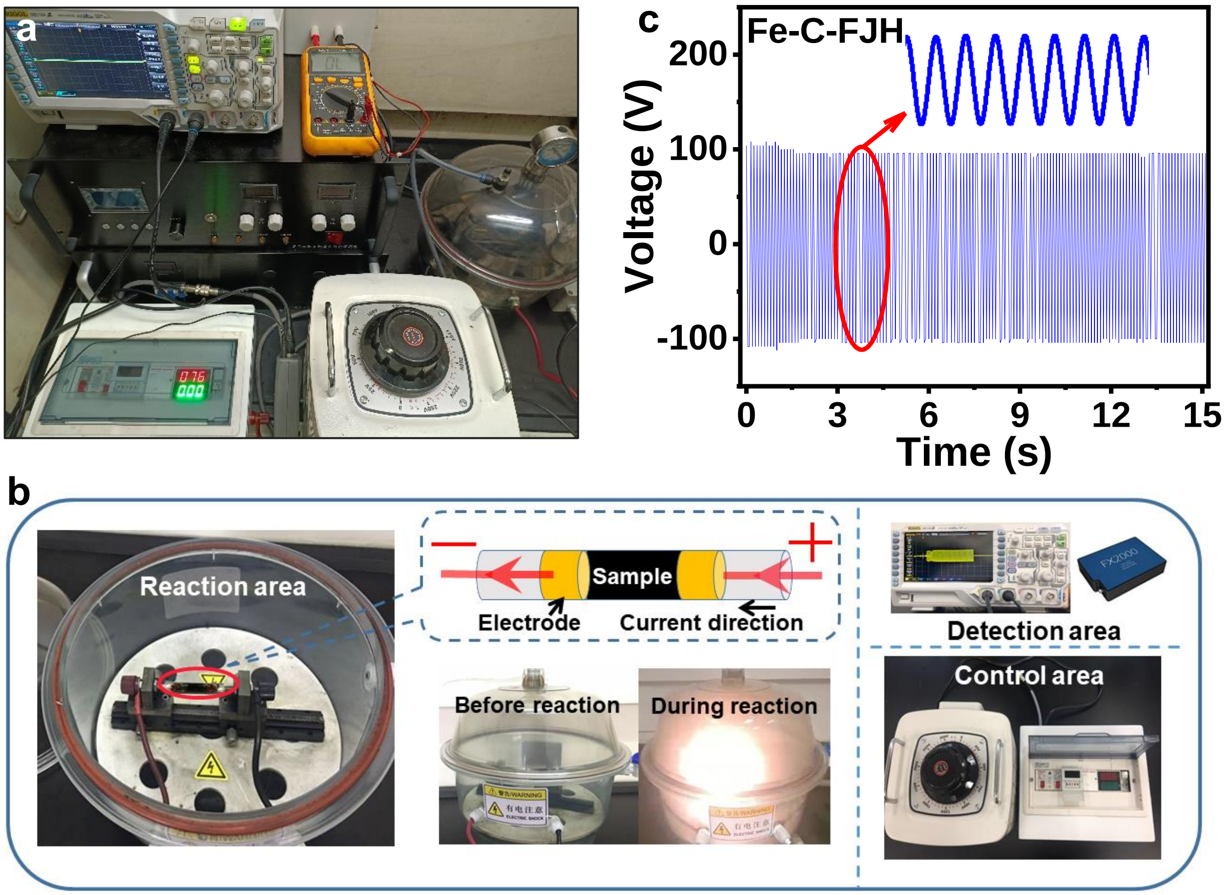

Supplementary Figure 1 I a Home-made FJH device. **b** The detailed parts of a homemade FJH device include a reaction area for putting samples, a detection area for recording voltage, current, temperature, and a control area for regulating preparation parameters. **c** Voltage was recorded in the FJH process. Note: The sample was passed through a current and a rapid thermal shock was induced to elevate the structures of sample in FJH process.

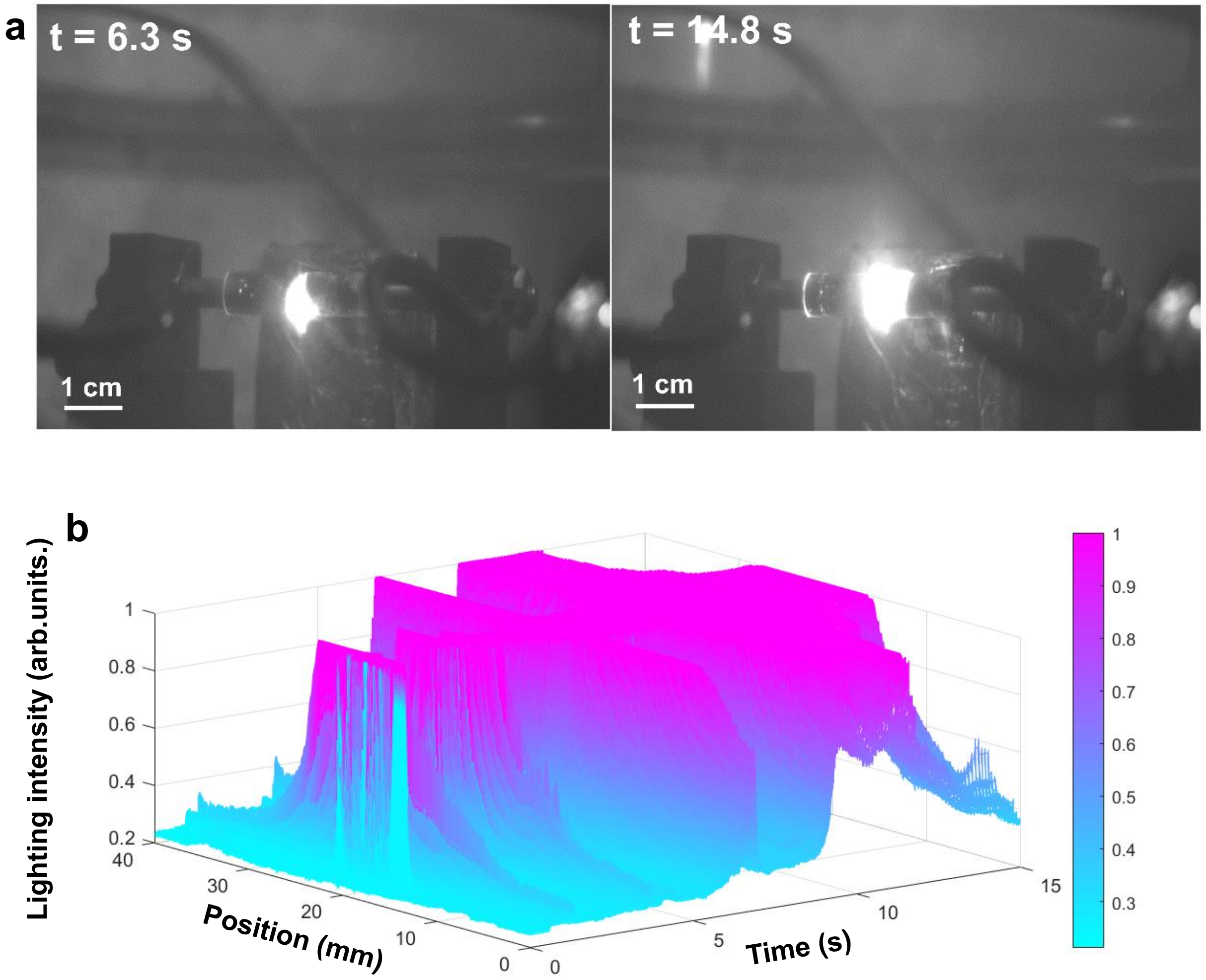

Supplementary Figure 2 I a Reacted phenomenon of different times recorded by a high-speed camera in the FJH process. **b** Lighting intensity of different reacted positions and time. Note: position represents the length of quartz tube in the reacted area.

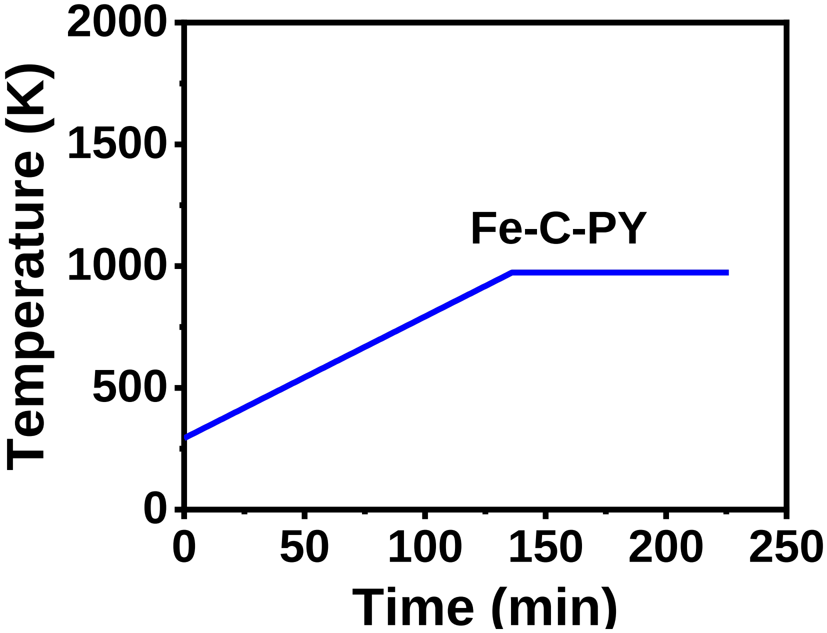

Supplementary Figure 3 l Temperature was recorded in the pyrolysis process.

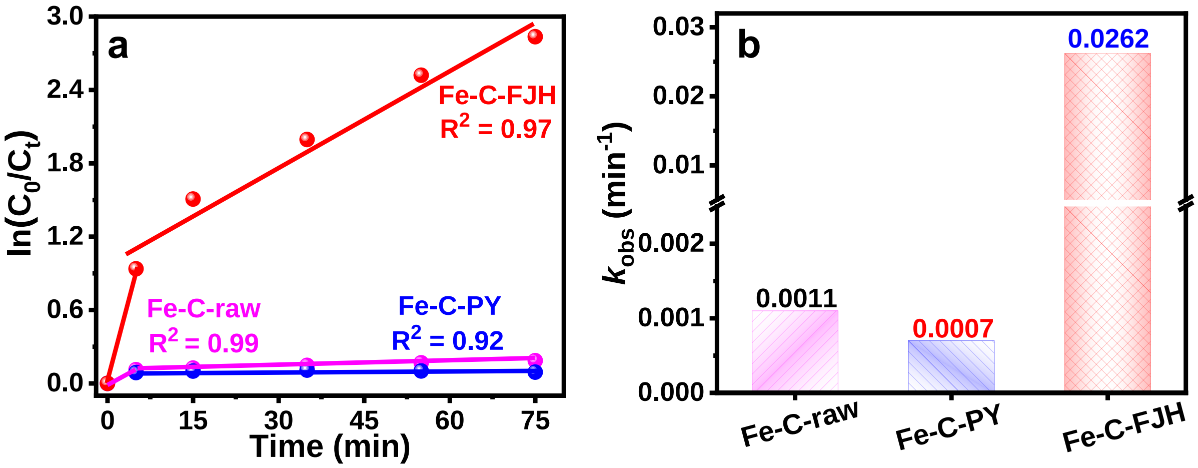

Supplementary Figure 4 I Kinetic analysis of catalytic CAP degradation. **a-b**, (**a**) $\ln(C_0/C_t)$ of CAP versus time, and (**b**) k_{obs} of CAP degradation by different Fe-based materials. Degradation condition: $[CAP]_0$ = 60 mg L^{-1}, [material] = 1000 mg L^{-1}, [PDS] = 7 mmol L^{-1}, initial pH_0 = 3.0±0.2, temperature = 28 °C.

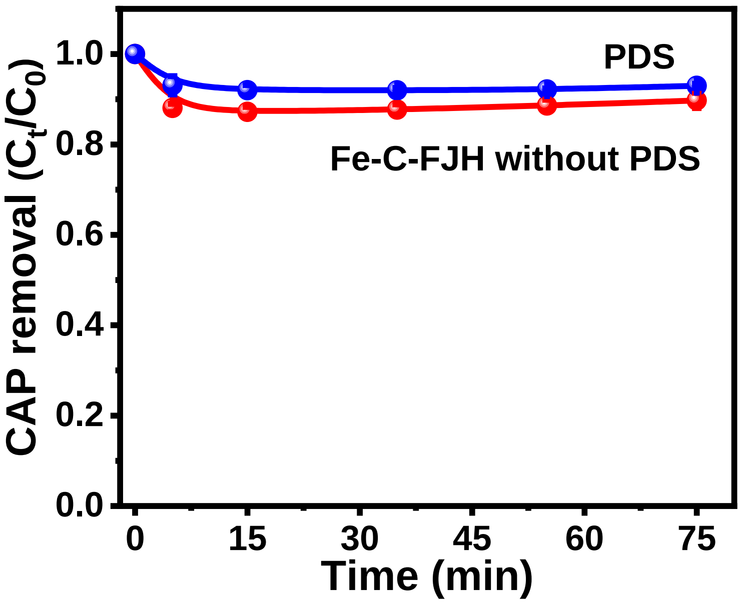

Supplementary Figure 5 I CAP removal by PDS and Fe-C-FJH independently. The experiments were repeated twice. Degradation condition: $[CAP]_0$ = 60 mg L^{-1}, [material] = 1000 mg L^{-1} or [PDS] = 7 mmol L^{-1}, initial pH_0 = 3.0±0.2, temperature = 28 °C.

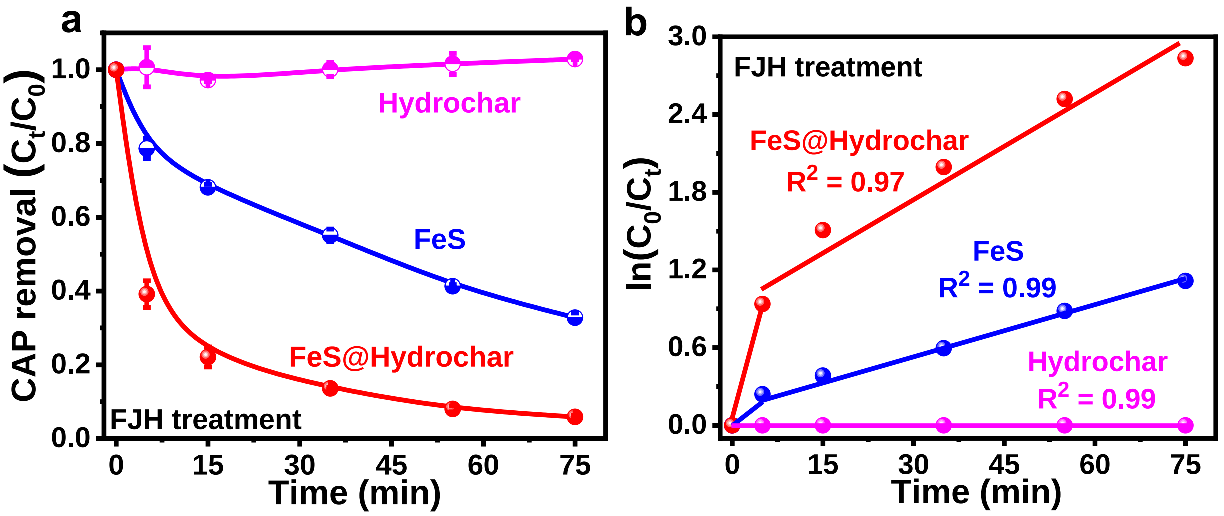

Supplementary Figure 6 l The effects of carbon substrate on catalytic performance. **a-b**, (**a**) CAP degradation and (**b**) $ln(C_0/C_t)$ of CAP versus time by different catalytic materials. Note: individual hydrochar, individual FeS, and the mixture of hydrochar and FeS were treated by FJH, respectively. The experiments were repeated twice (a). Degradation condition: $[CAP]_0$ = 60 mg L^{-1}, [material] = 1000 mg L^{-1}, [PDS] = 7 mmol L^{-1}, initial pH_0 = 3.0±0.2, temperature = 28 ^oC.

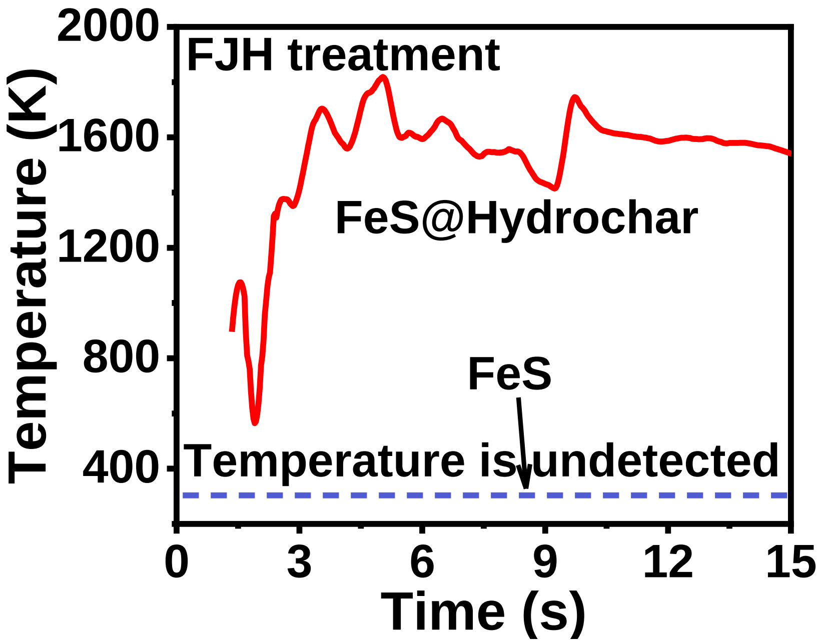

Supplementary Figure 7 I Temperature variations during FJH process. Note: individual hydrochar and the mixture of hydrochar and FeS were treated by FJH, respectively.

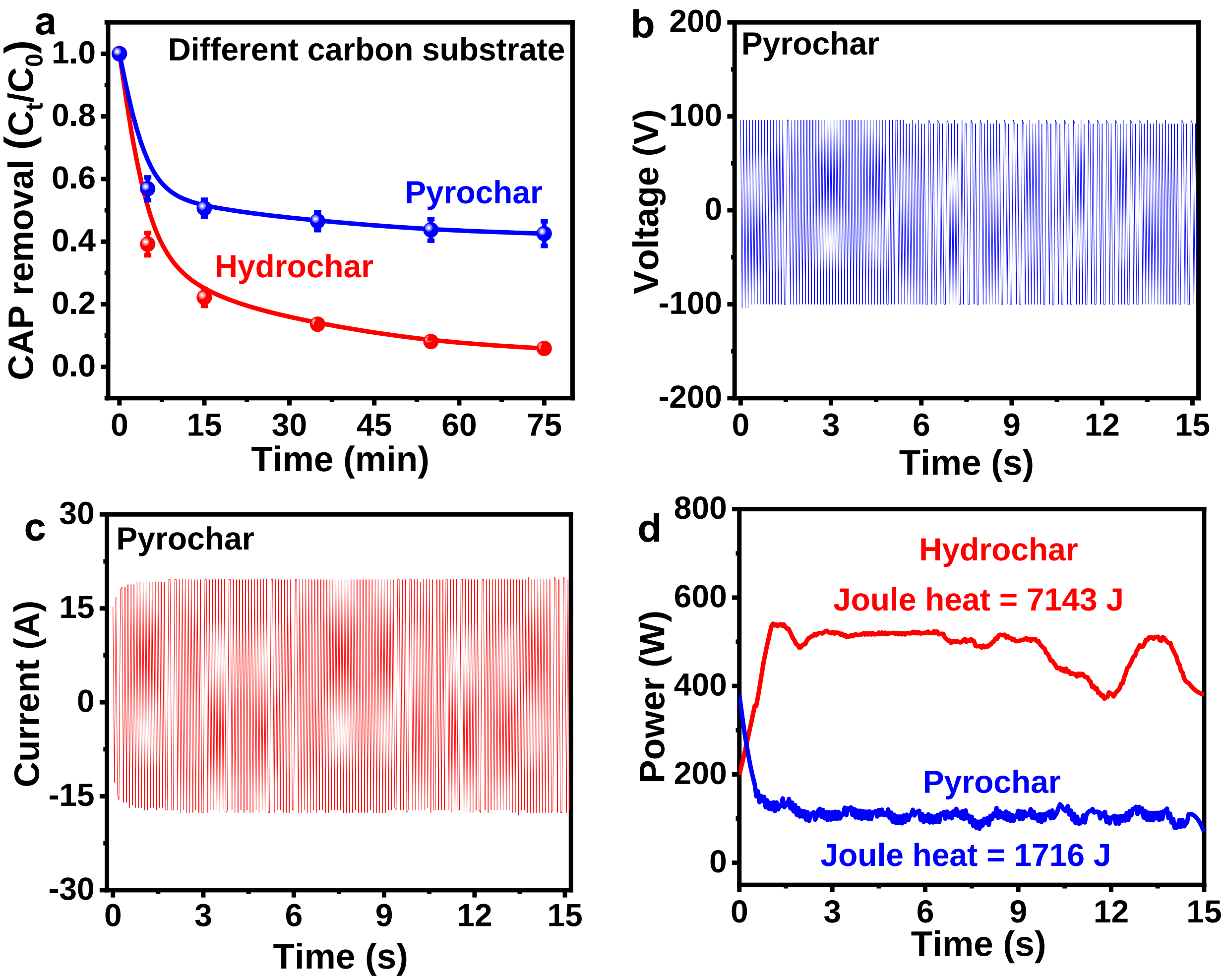

Supplementary Figure 8 I a CAP degradation by the Fe-based material prepared with different carbon substrates in FJH process. The experiments were repeated twice. Degradation condition: $[CAP]_0$ = 60 mg L^{-1}, [material] = 1000 mg L^{-1}, [PDS] = 7 mmol L^{-1}, initial pH_0 = 3.0±0.2, temperature = 28 °C. **b-c**, (**b**) Voltage and (**c**) current were recorded in FJH process. **d** The power and Joule heat were produced in FJH processes with different carbon substrates. Note: Power was acquired by multiplying voltage and current, and Joule heat was acquired by multiplying power and time.

Supplementary Table 1 I The comparison of CAP degradation efficiency by different Fe-based materials. Normalized removal was evaluated according to that the CAP removal mass divided unit Fe-based material mass and time.

Samples	Pollutant concentration	Reacted condition	Normalized removal (mg $(g\cdot min)^{-1}$)	References
nFe (ZVI)	10 mg L^{-1}	PDS = 0.2 mM, material = 0.5 g L^{-1}, pH = 7.0, time = 120 min	0.119	[14]
α-FeOOH	20 mg L^{-1}	PDS = 20 mg L^{-1}, a-FeOOH = 5.6 mM, pH = 3 time = 100 min	0.402	[15]
nFe (ZVI)	5 mg L^{-1}	PDS = 1 mM, Fe = 1000 mg L^{-1}, time = 90 min, Ultrasonic intensity = 0.36 W mL^{-1}	0.056	[16]
Bi2SeO5/rGO/MIL-88A	5 mg L^{-1}	material = 300 mg L^{-1}, pH = 6.12 time = 100 min Light intensity = 300 W	0.119	[17]
Fe-C-FJH	60 mg L^{-1}	PDS = 7 mM, Fe = 1000 mg L^{-1}, pH = 2.8, time = 35 min	1.481	This work

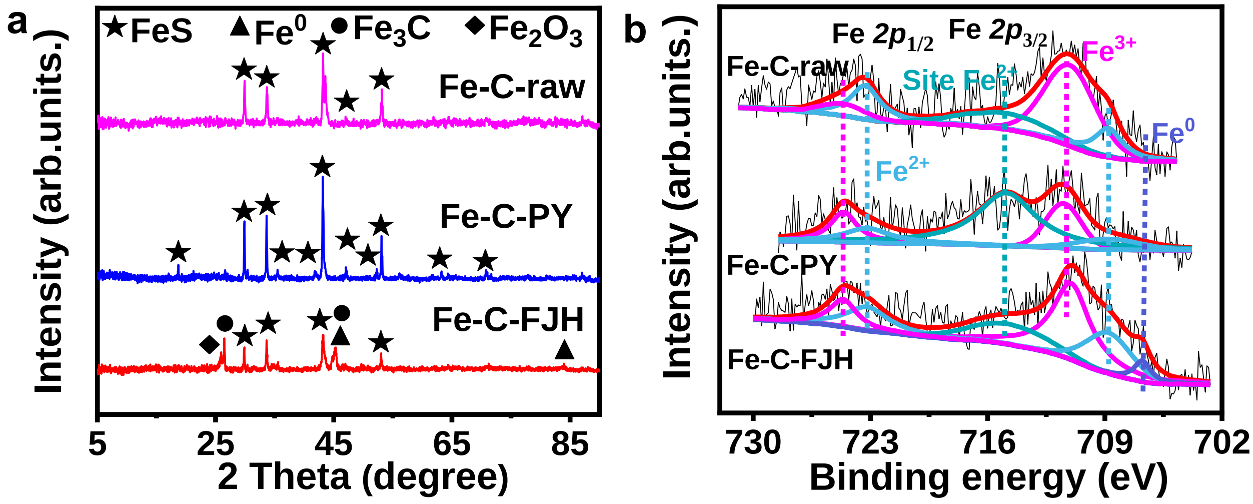

Supplementary Figure 9 I a-b, (**a**) XRD patterns and (**b**) Fe *2p* XPS spectra of the Fe-based material prepared by FJH and conventional pyrolysis.

Supplementary Table 2 I Fe components of the Fe-based material prepared by FJH and conventional pyrolysis, detected by ^{57}Fe Mössbauer spectroscopy.

Sample	Site	H_f (KOe)	IS (mm s^{-1})	QS (mm s^{-1})	Γ/2 (mm s^{-1})	Content (%)
Fe-C-PY	FeS	310.6	0.76	0.14	0.18	100
Fe-C-FJH	FeS	310.12	0.59	-0.15	0.21	26.49
	Fe_7S_8	292.55	-0.01	0.14	0.33	24.98
	Fe^0	324.44	0.00	-0.15	0.36	14.94
	Fe_3C	204.25	-0.08	-0.01	0.37	29.32
	Fe_2O_3	-	0.17	0.91	0.16	4.27

Note: H_f = hyperfine field, IS = isomer shift, QS = quadrupole splitting, Γ/2 = half band width.

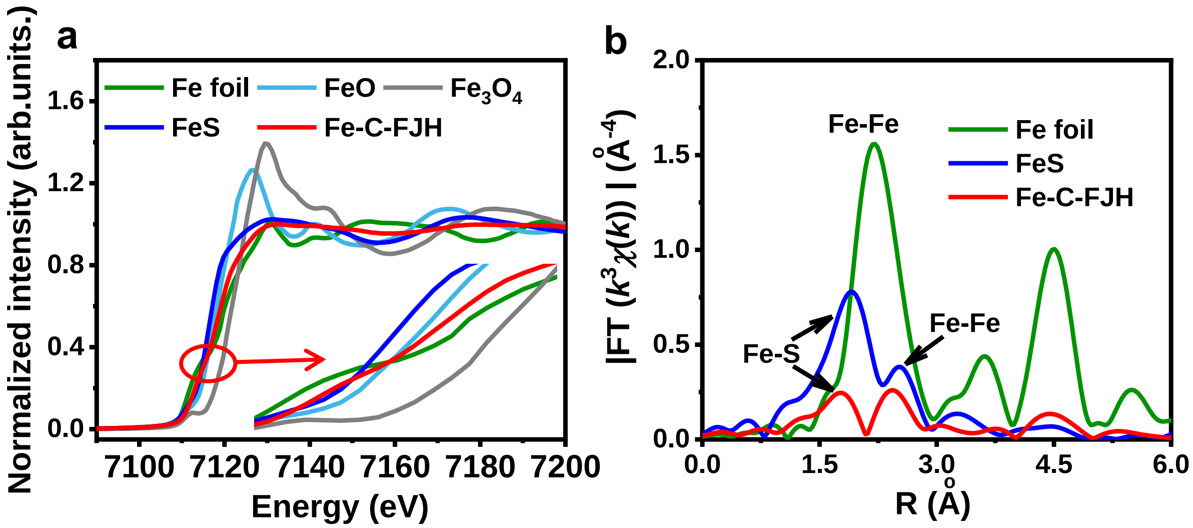

Supplementary Figure 10 I a-b, (**a**) Fe-XANES K-edge spectra and (**b**) the k^3-weighted Fourier transform spectra from Fe K-edge EXAFS of the Fe-based material prepared by FJH.

Supplementary Table 3 I EXAFS fitting parameters at the Fe K-edge for various samples.

Sample	Shell	CN^a	R (Å)^b	σ^2 (Å^2)^c	ΔE_0 (eV)^d	R factor
Fe foil	Fe-Fe	8*	2.47±0.01	0.0045±0.0008	7.3	0.0016
	Fe-Fe	6*	2.86±0.01	0.0063±0.0016		
FeS	Fe-S	3.9±0.5	2.41±0.01	0.0112±0.0016	2.9	0.0035
	Fe-Fe	3.7±1.9	2.93±0.01	0.0179±0.0063		
Fe-C-FJH	Fe-S	1.7±0.2	2.36±0.01	0.0075±0.0015	9.5	0.0200
	Fe-Fe	1.7±0.4	2.46±0.02	0.0101±0.0014		

Note: ^{a}CN, coordination number; ^{b}R, the distance between absorber and backscatter atoms; $^c\sigma^2$, Debye-Waller factor to account for both thermal and structural disorders; $^d\Delta E_0$, inner potential correction; R factor indicates the goodness of the fitting; * represents the fixing CN of Fe foil.

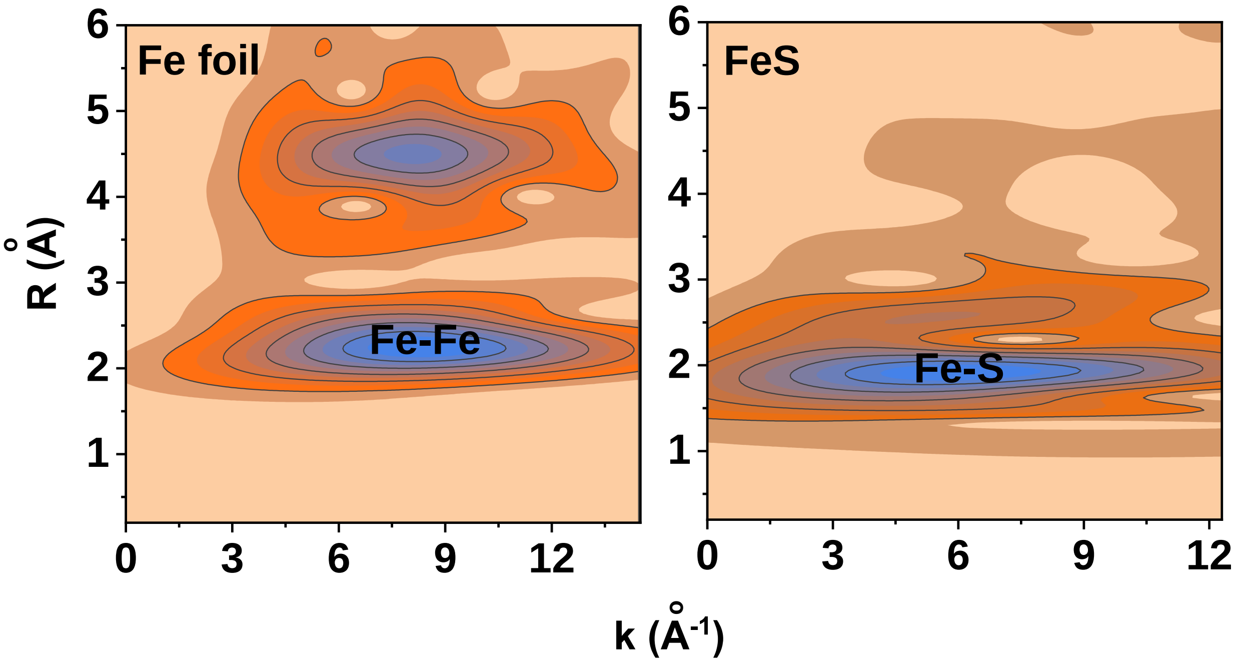

Supplementary Figure 11 I Wavelet transform analysis of Fe foil and FeS standards.

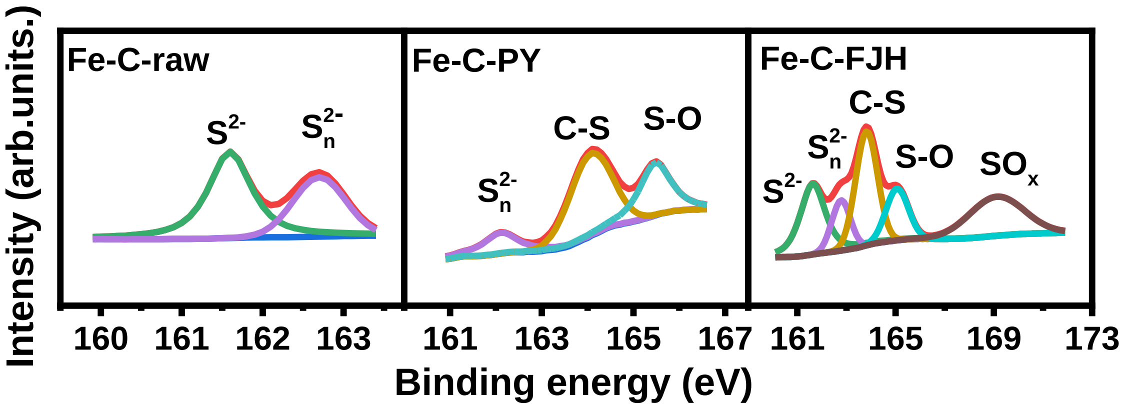

Supplementary Figure 12 I S *2p* XPS spectra of the Fe-based material prepared by FJH and conventional pyrolysis.

Supplementary Table 4 I The elemental analysis of the Fe-based material prepared by FJH and conventional pyrolysis.

Samples	C (%)	H (%)	N (%)	S (%)	O (%)	H/C	Ash (%)
Fe-C-raw	39.69	1.26	0.96	13.15	17.41	0.38	40.68
Fe-C-PY	40.66	0.21	0.73	12.56	13.88	0.06	44.52
Fe-C-FJH	35.69	0.16	0.39	10.19	11.83	0.05	51.93

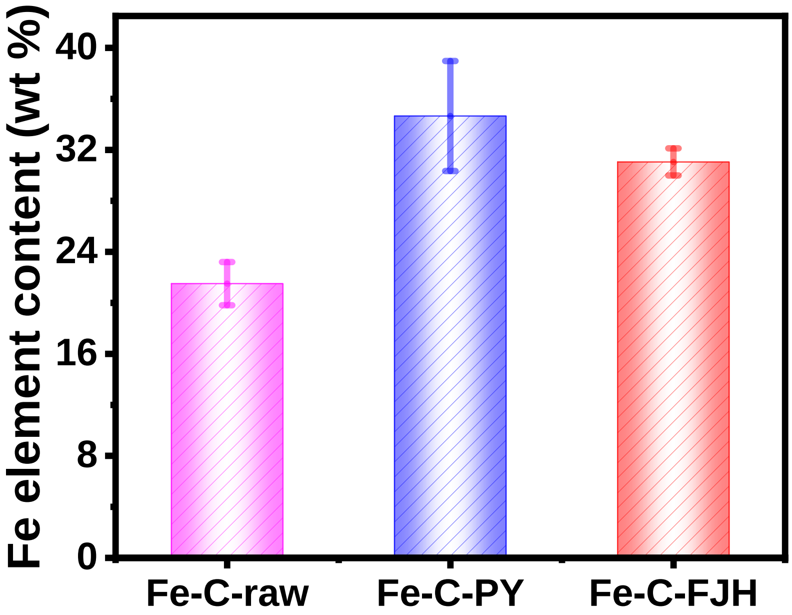

Supplementary Figure 13 I Fe element contents of the Fe-based material prepared by FJH and conventional pyrolysis by ICP measurements. The experiments were repeated twice.

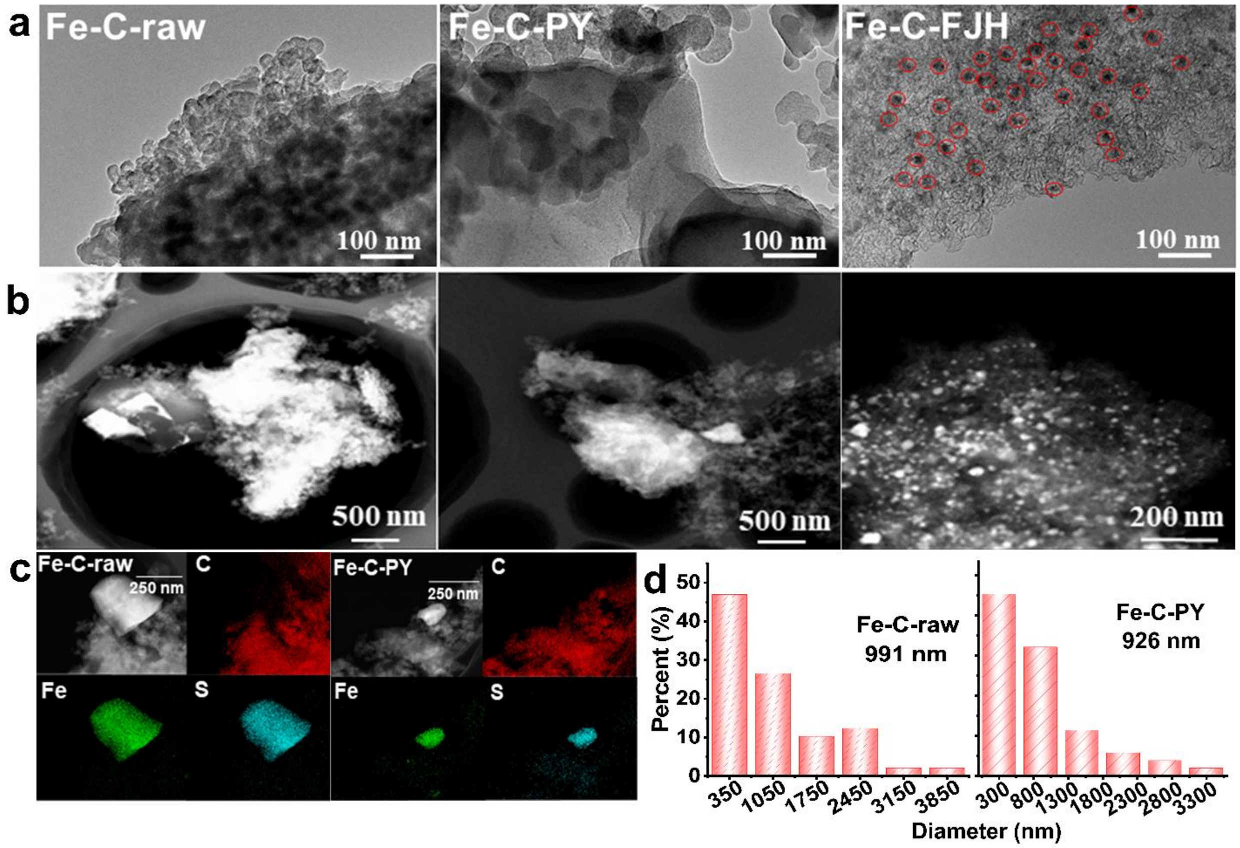

Supplementary Figure 14 I a-b, (**a**) TEM and (**b**) HAADF-TEM images of the Fe-based material prepared by FJH and conventional pyrolysis. **c** TEM with EDS elemental mappings of C, Fe, and S elements distribution in different Fe-based materials. **d** The Fe composite particle size of Fe-C-FJH.

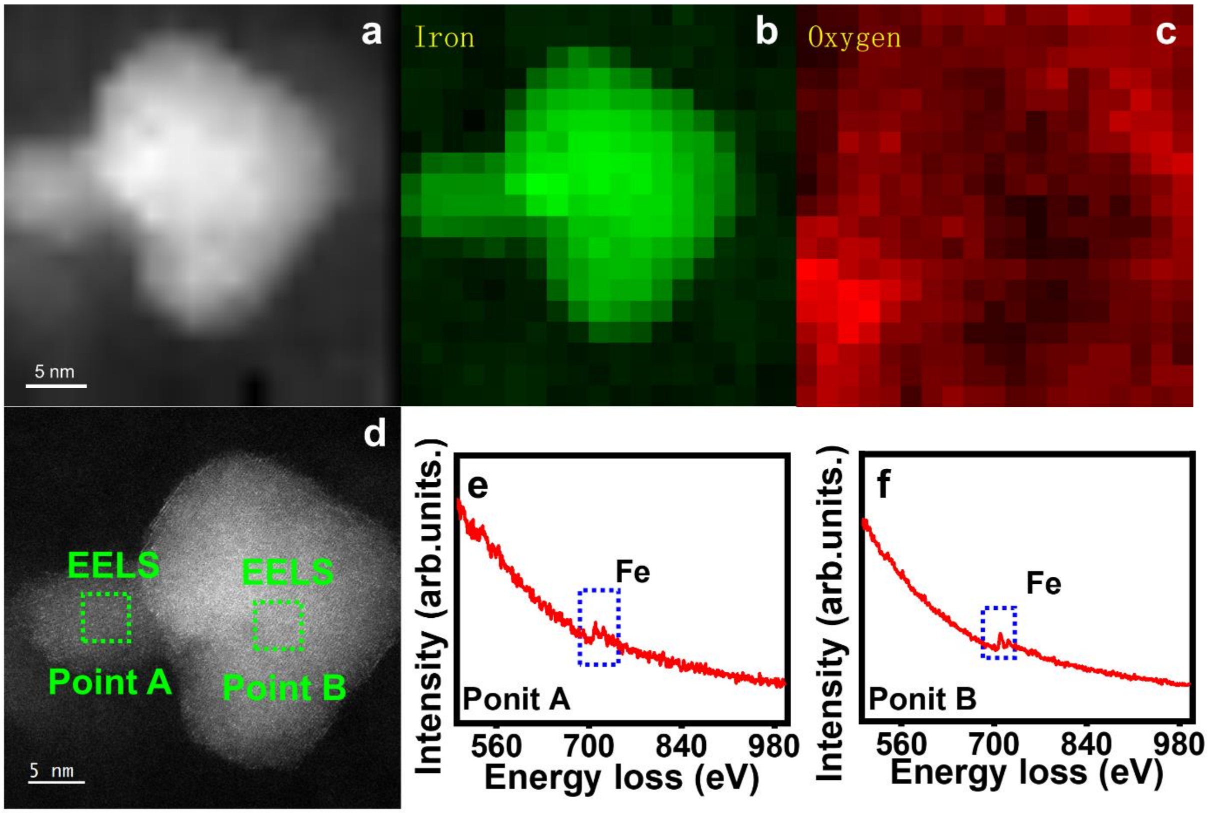

Supplementary Figure 15 I a-c, HAADF-STEM image and EDS mapping of Fe and O elements distribution in Fe-C-FJH. **d-f,** Fe L_3 STEM-EELS data from point A and point B.

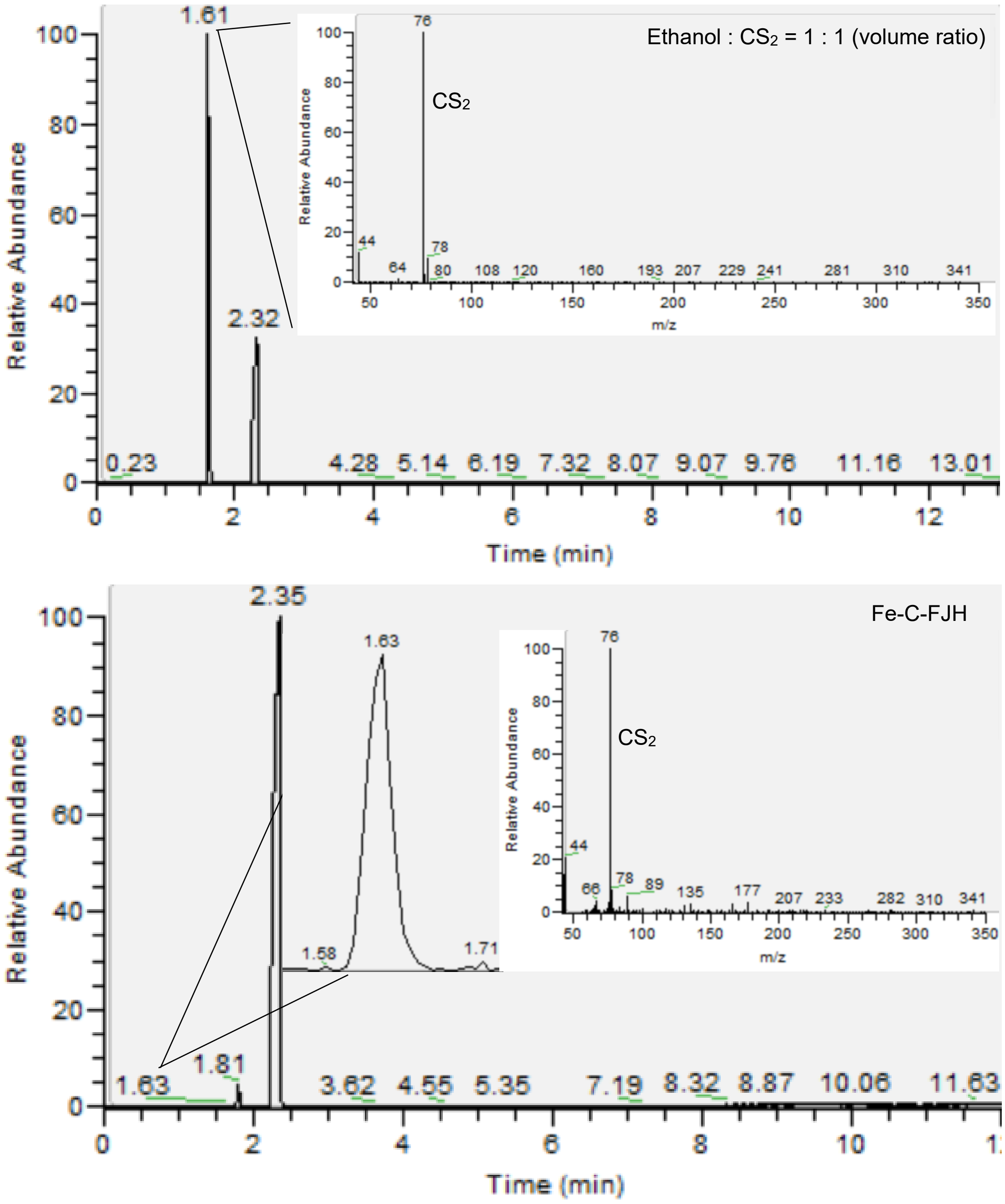

Supplementary Figure 16 I The CS_2 detection by ethanol collection liquid from the FJH process.

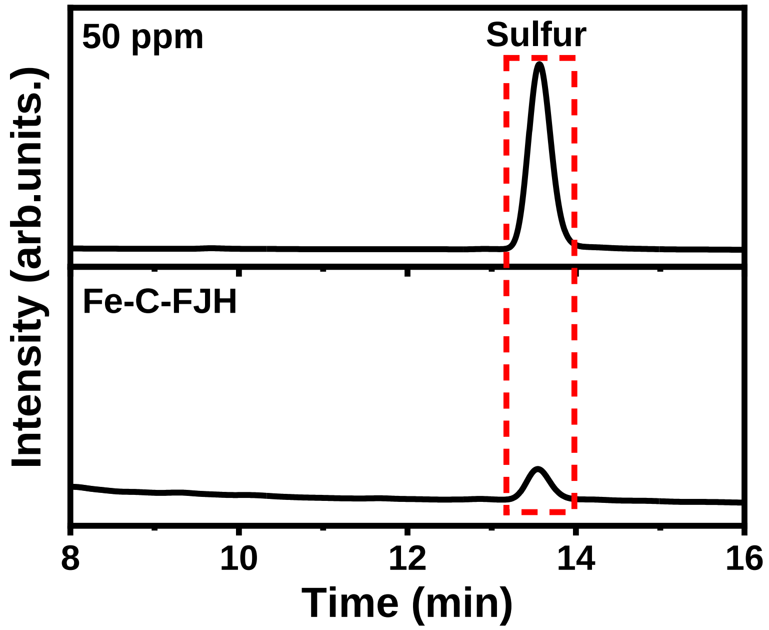

Supplementary Figure 17 I The sulfur peak were detected by HPLC from sulfur standard and Fe-C-FJH.

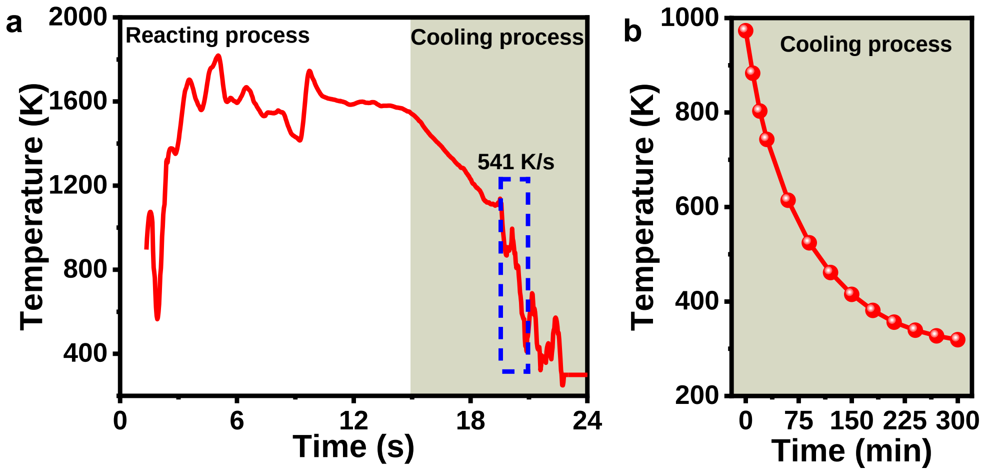

Supplementary Figure 18 I a-b, The temperatures were recorded in the (**a**) FJH and (**b**) pyrolysis cooling processes for Fe-C-FJH and Fe-C-PY preparation.

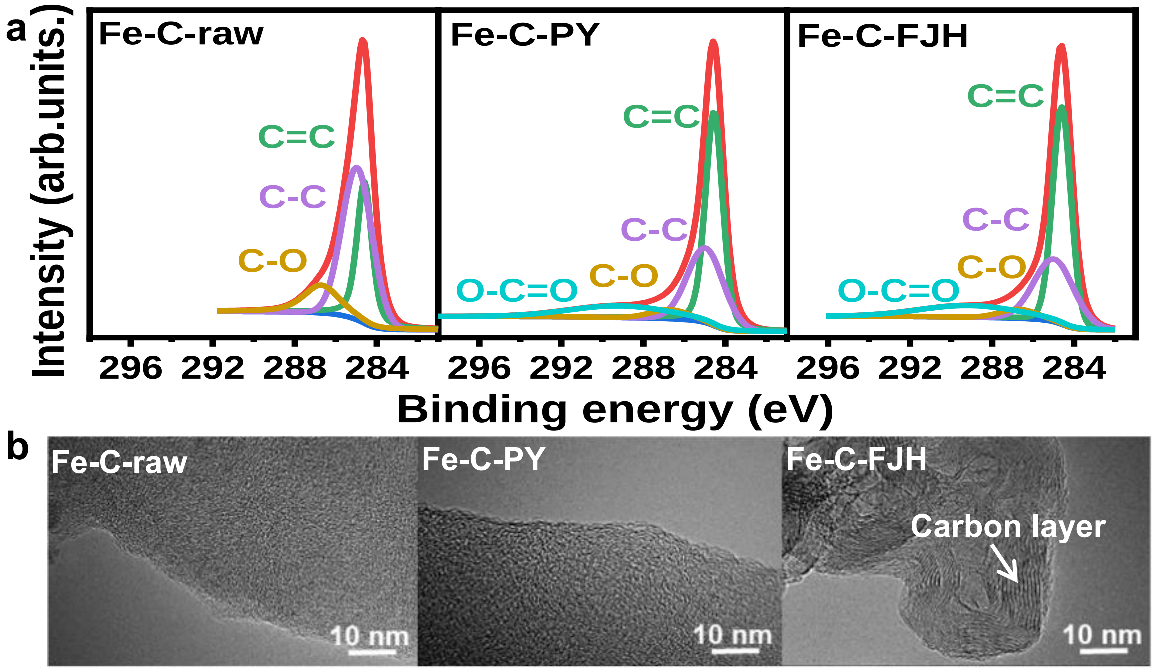

Supplementary Figure 19 I a-b, (**a**) C *1s* XPS spectra and (**b**) HRTEM images of the Fe-based material prepared by FJH and conventional pyrolysis.

Supplementary Table 5 I The graphitization degree of the Fe-based material prepared by FJH and conventional pyrolysis by Raman and C *1s* XPS measurements.

Samples	I_G/I_D	I_{2D}/I_G	sp^2/sp^3
Fe-C-raw	0.31	-	0.57
Fe-C-PY	0.49	-	1.53
Fe-C-FJH	0.87	0.81	1.69

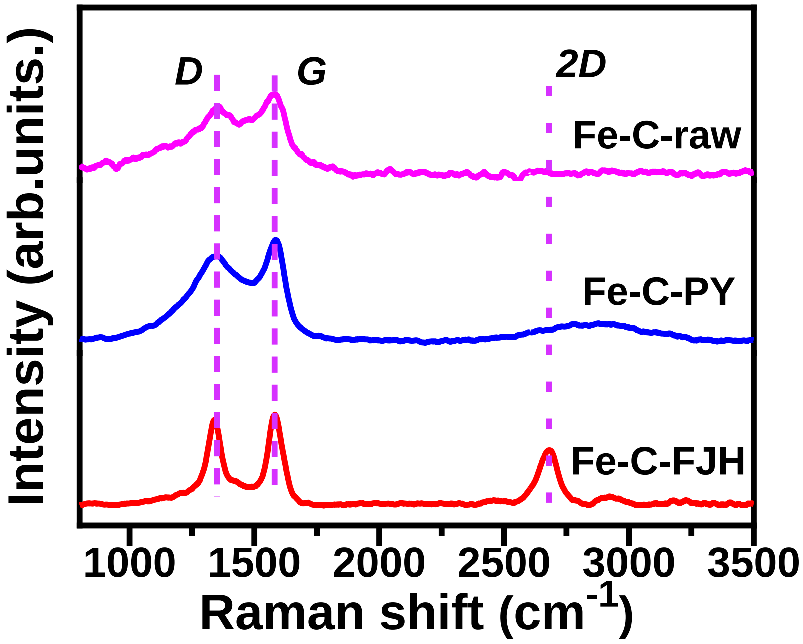

Supplementary Figure 20 I Raman spectra of Fe-C-raw, Fe-C-PY, and Fe-C-FJH.

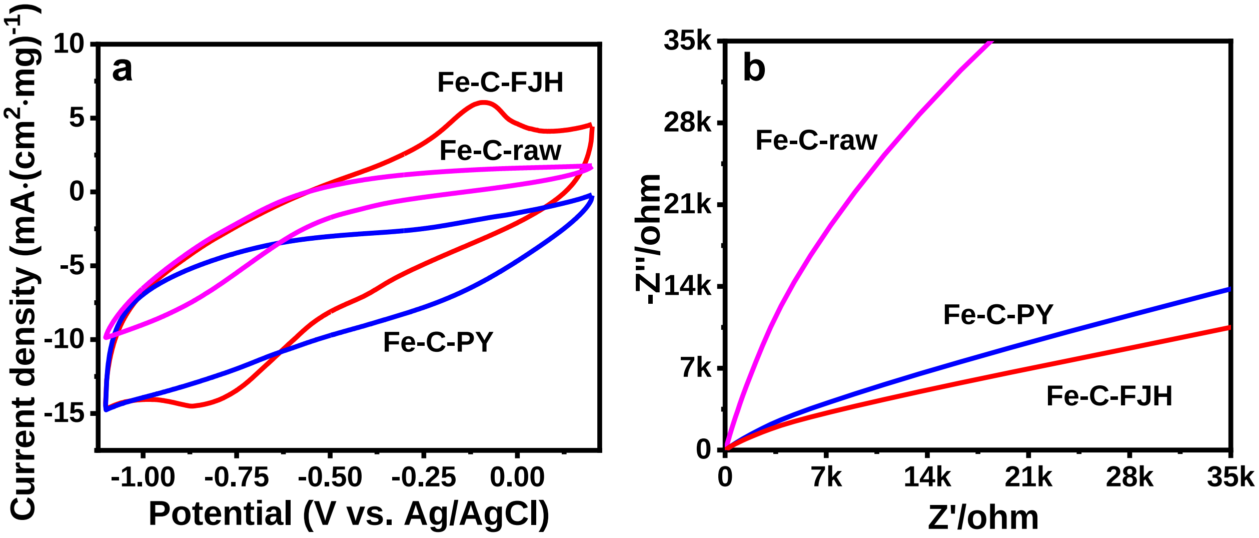

Supplementary Figure 21 I a-b, (**a**) cyclic voltammetry and (**b**) electrochemical impedance spectroscopy curves of the Fe-based material prepared by FJH and conventional pyrolysis.

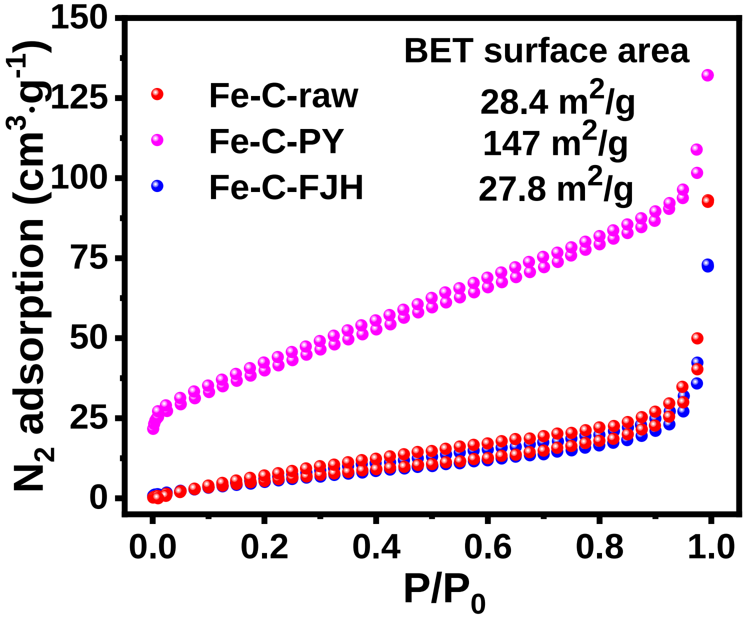

Supplementary Figure 22 I N_2 adsorption and desorption curves, and surface areas of the Fe-based material prepared by FJH and conventional pyrolysis.c

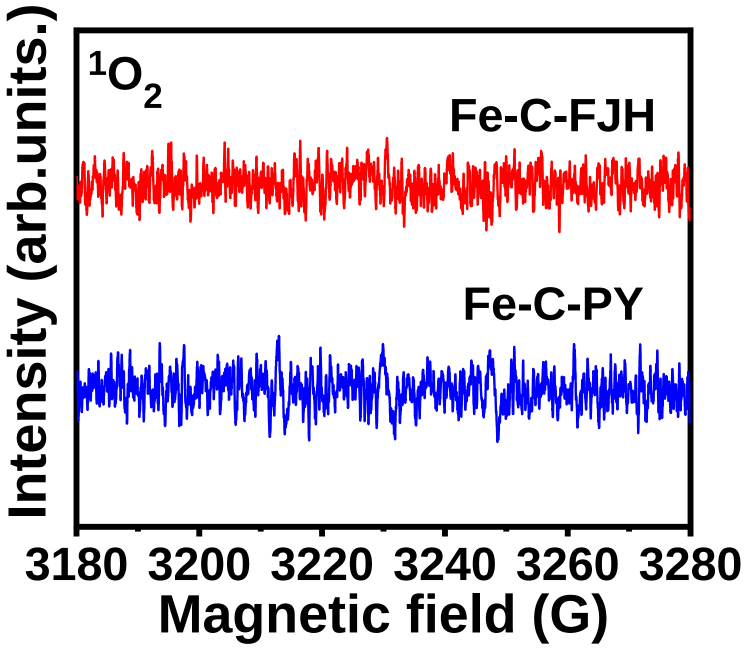

Supplementary Figure 23 I EPR spectra of Fe-based material prepared by FJH and conventional pyrolysis, were tested by using TEMP as trapping agents for $^1O_2$.

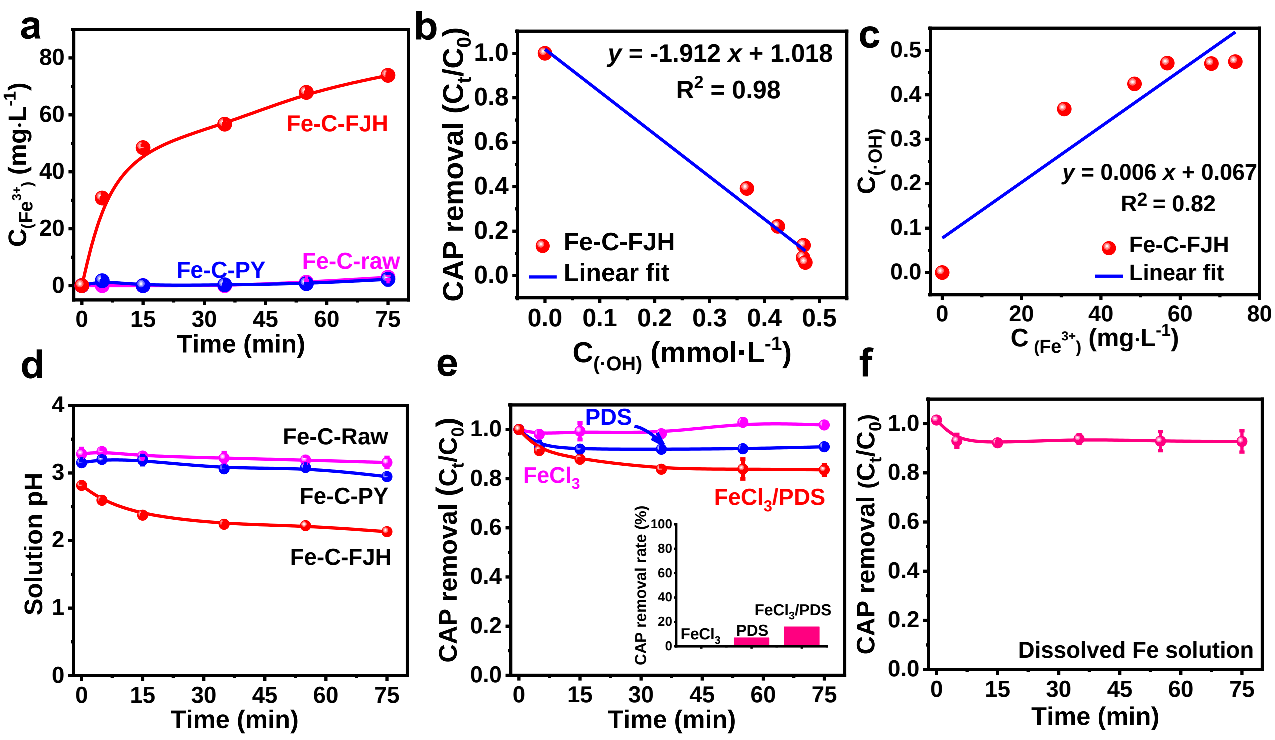

Supplementary Figure 24 I Transformation of Fe species in the degradation process. **a** Dissolved Fe^{3+} concentration in the degradation process. **b** The linear relationships between the concentration of Fe^{3+} and CAP degradation efficiency. **c** The linear relationship between the concentrations of ·OH and Fe^{3+}. **d** The change of solution pH during the CAP removal process. $[CAP]_0$ = 60 mg L^{-1}, [material] = 1000 mg L^{-1}, [PDS] = 7 mmol L^{-1}, initial pH_0 = 3.0±0.2, temperature = 28 °C. **e** The effect of Fe^{3+} on CAP removal, and CAP removal rate at 75 min was put in the inset image. Degradation condition: $[CAP]_0$ = 60 mg L^{-1}, $[FeCl_3]$ = 1000 mg L^{-1} (if need), [PDS] = 7 mmol L^{-1} (if need), initial pH_0 = 3.0±0.2, temperature = 28 °C. **f** CAP removal of dissolved Fe solution from the finished system of Fe-C-FJH/PDS/CAP at 75 min by adding the new PDS and CAP components. The experiments were repeated twice (d, e, and f).

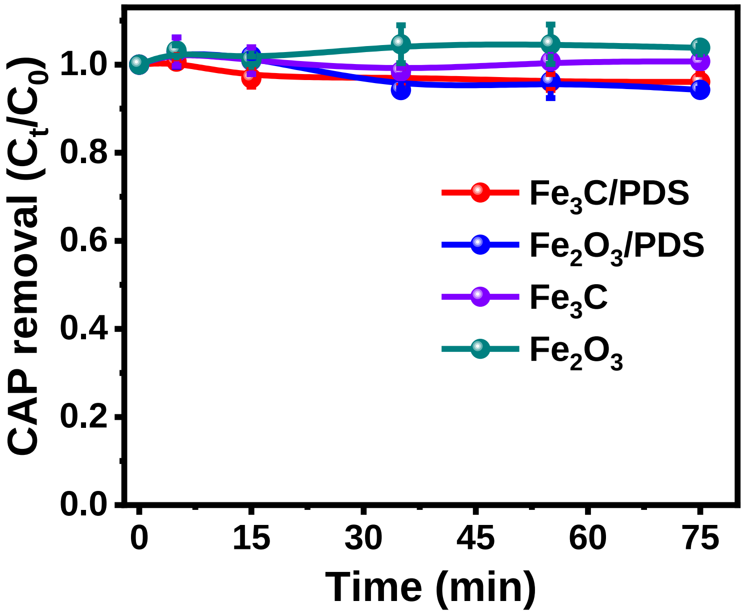

Supplementary Figure 25 I The CAP removal of commercial Fe_3C and Fe_2O_3. The experiments were repeated twice. Degradation condition: $[CAP]_0$ = 60 mg L^{-1}, [PDS] = 7 mmol L^{-1} (if need), initial pH_0 = 3.0±0.2, temperature = 28 °C. The additive amount of commercial Fe_3C and Fe_2O_3 was the same as the corresponding Fe content of Fe-C-FJH according to the ICP and Fe Mössbauer spectra results.

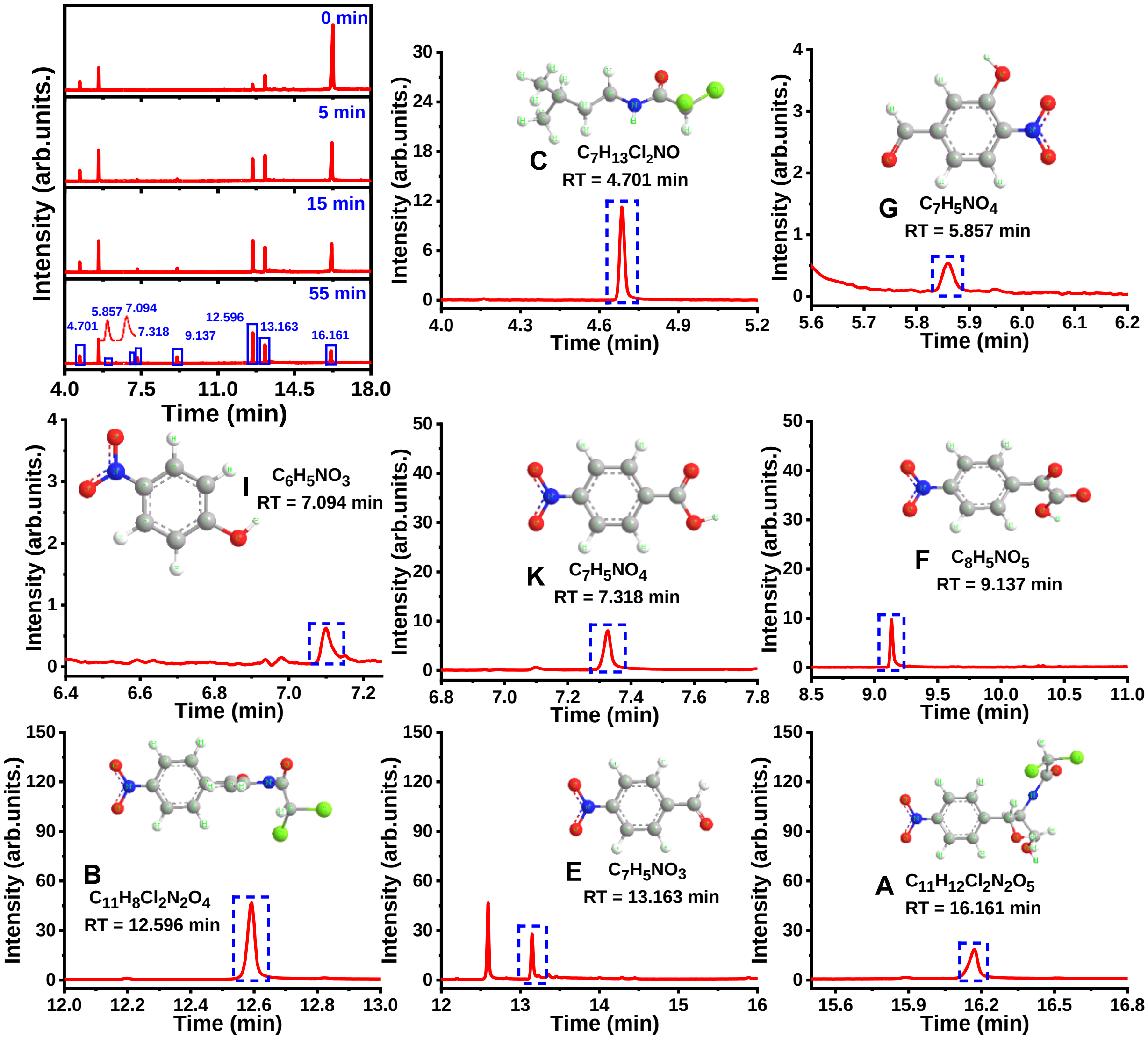

Supplementary Figure 26 I Intermediate products of CAP degradation by Fe-C-FJH were measured from GC-MS. Degradation condition: $[CAP]_0$ = 300 mg L^{-1}, [material] = 2000 mg L^{-1}, [PDS] = 14 mmol L^{-1}, initial pH_0 = 3.0±0.2.

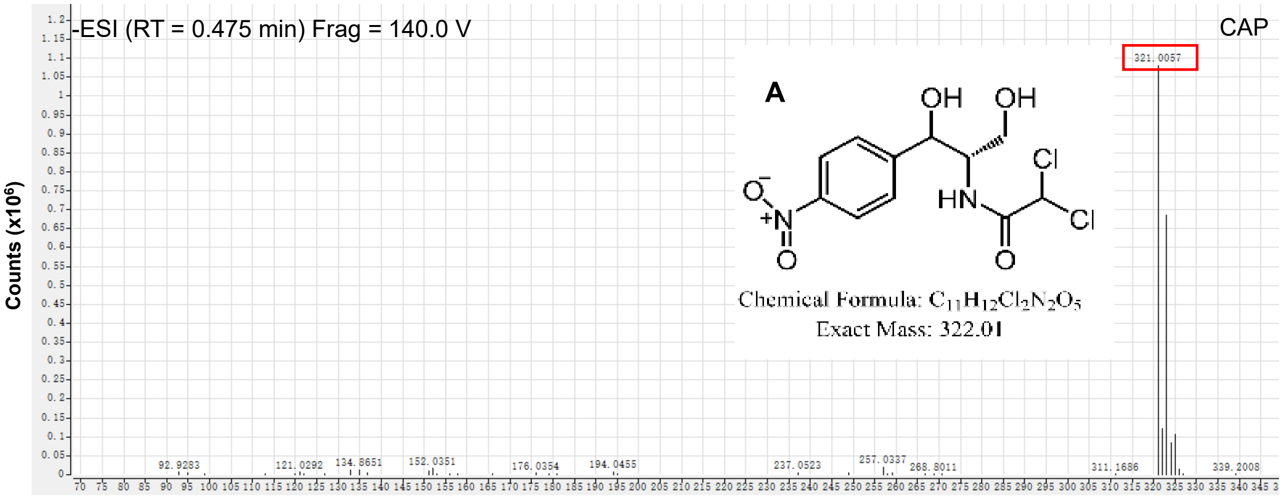
-ESI (RT = 0.475 min) Frag = 140.0 V
CAP
A
Chemical Formula: C11H12Cl2N2O5
Exact Mass: 322.01
321.0057
Counts (x10^6)

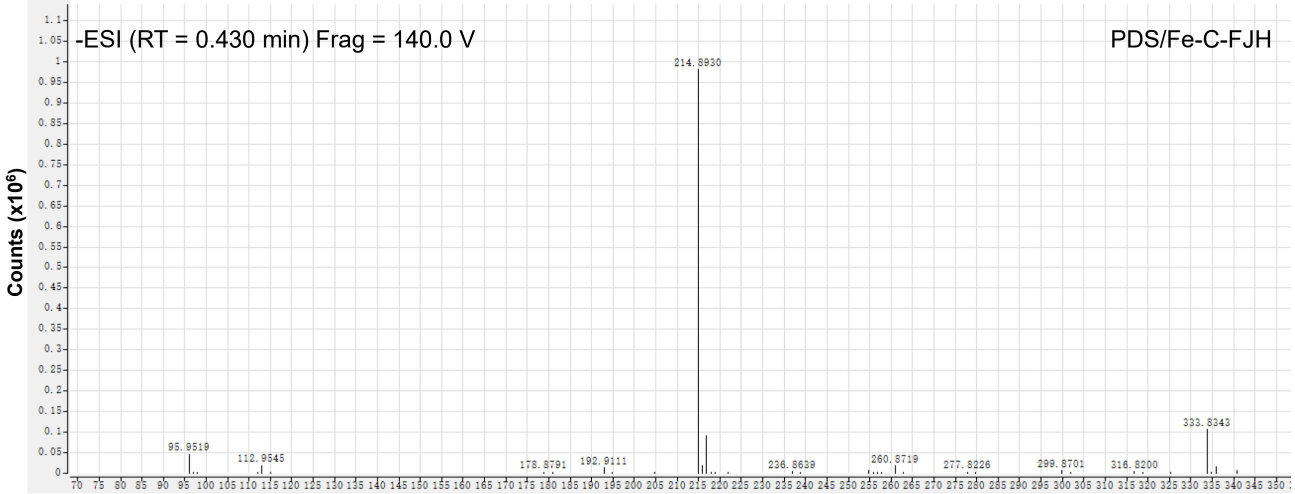
-ESI (RT = 0.430 min) Frag = 140.0 V
PDS/Fe-C-FJH
214.8930
333.8343
Counts (x10^6)

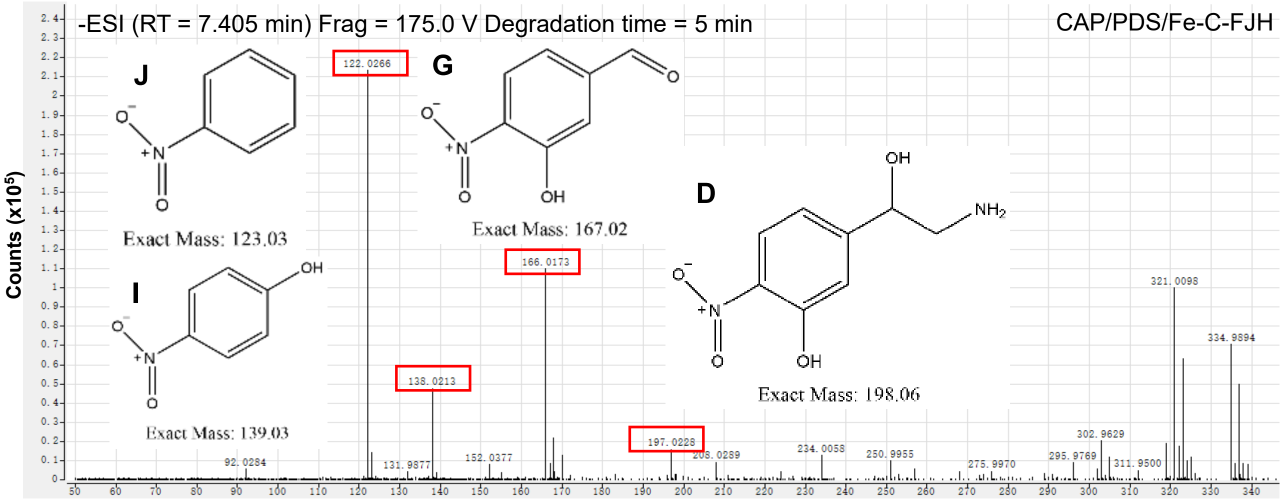
-ESI (RT = 7.405 min) Frag = 175.0 V Degradation time = 5 min
CAP/PDS/Fe-C-FJH
J
Exact Mass: 123.03
G
Exact Mass: 167.02
D
Exact Mass: 198.06
I
Exact Mass: 139.03
122.0266
166.0173
138.0213
197.0228
321.0098
334.9894
Counts (x10^5)

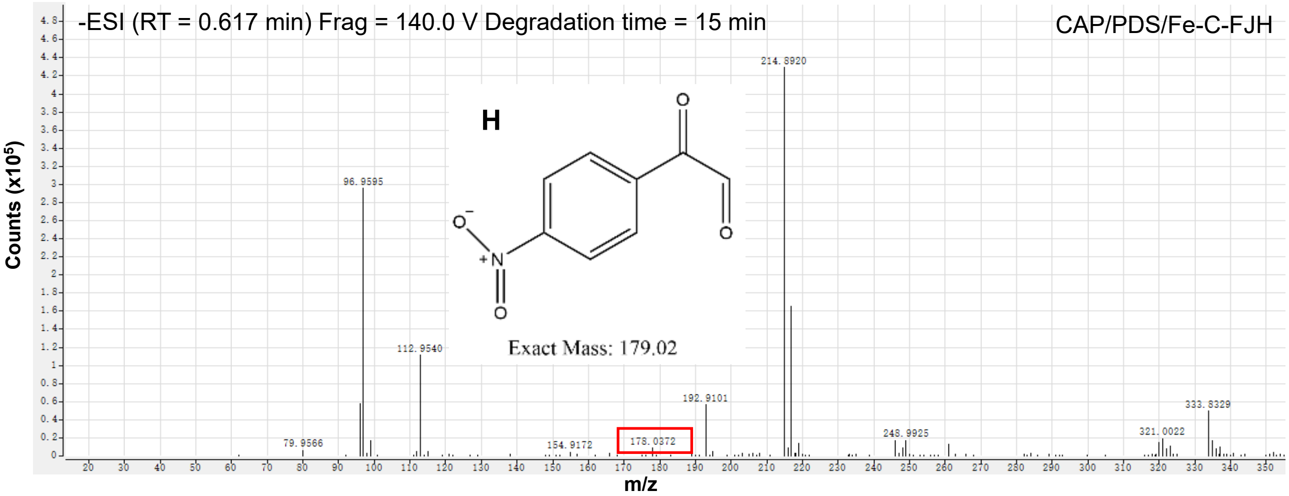

Supplementary Figure 27 I Intermediate products of CAP degradation by Fe-C-FJH were measured from HPLC/TOF-MS. Degradation condition: $[CAP]_0$ = 300 mg L^{-1}, [material] = 2000 mg L^{-1}, [PDS] = 14 mmol L^{-1}, initial pH_0 = 3.0±0.2.

Supplementary Table 6 I The concentrations of Cl^- and NO_3^- produced in the CAP degradation process by FJH.

Anions	Retention time	Concentration (μg L^{-1})
Cl^-	5.23	2799.4
NO_3^-	9.71	1794.6

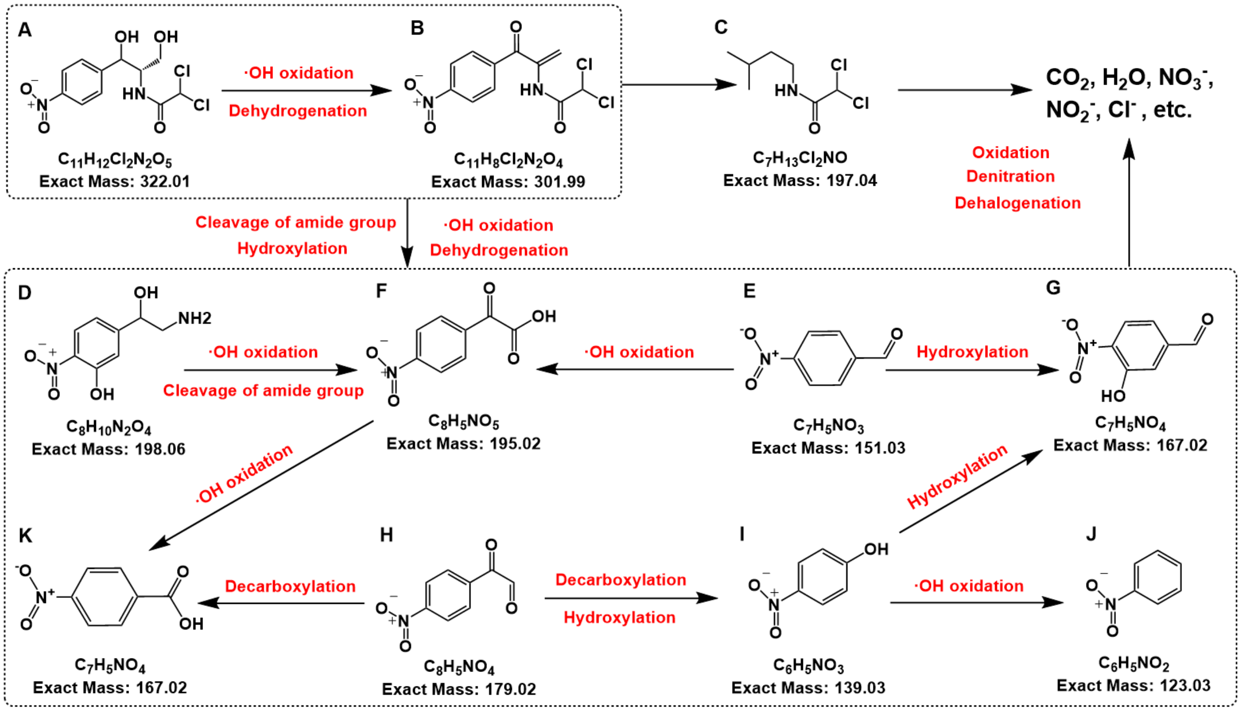

Supplementary Figure 28 I Proposed CAP degradation pathways from the system of CAP/PDS/Fe-C-FJH.

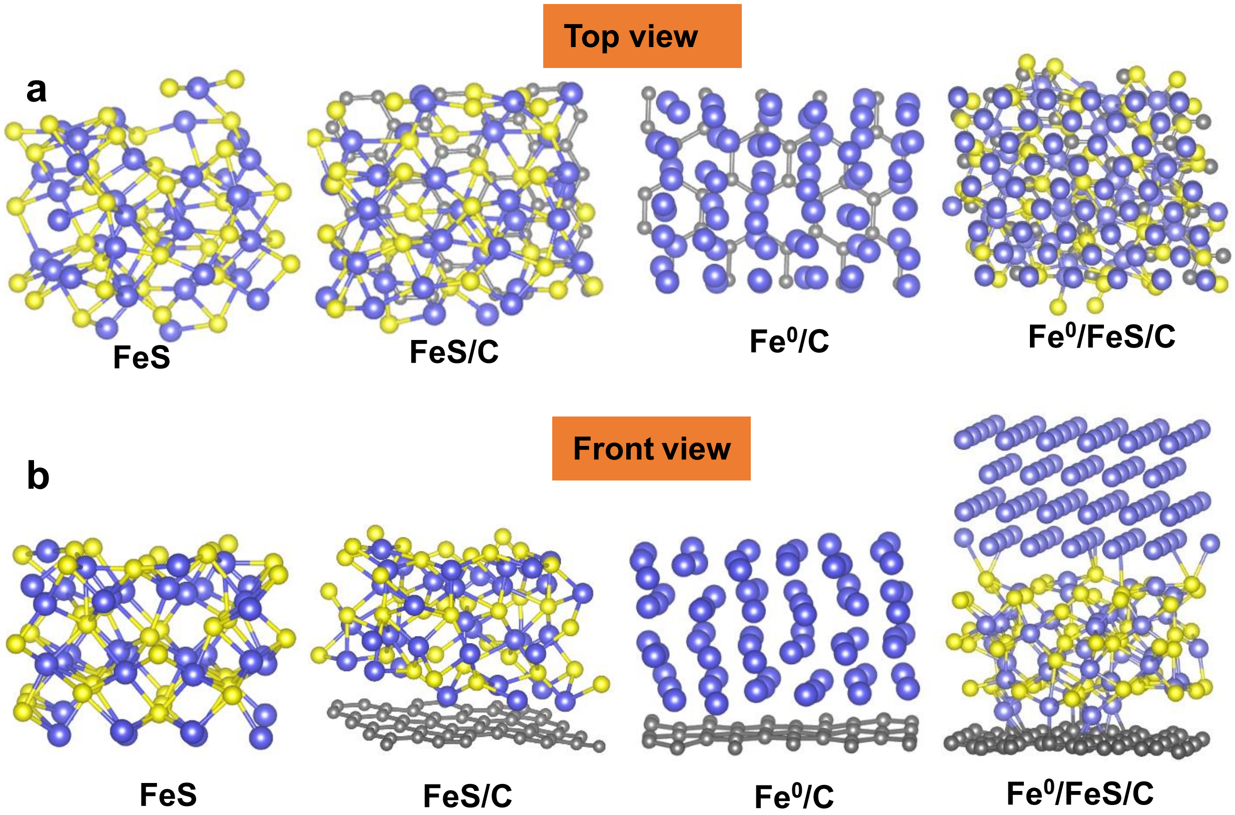

Supplementary Figure 29 I a-b, The optimized structures of different components in (**a**) top view and (**b**) front view.

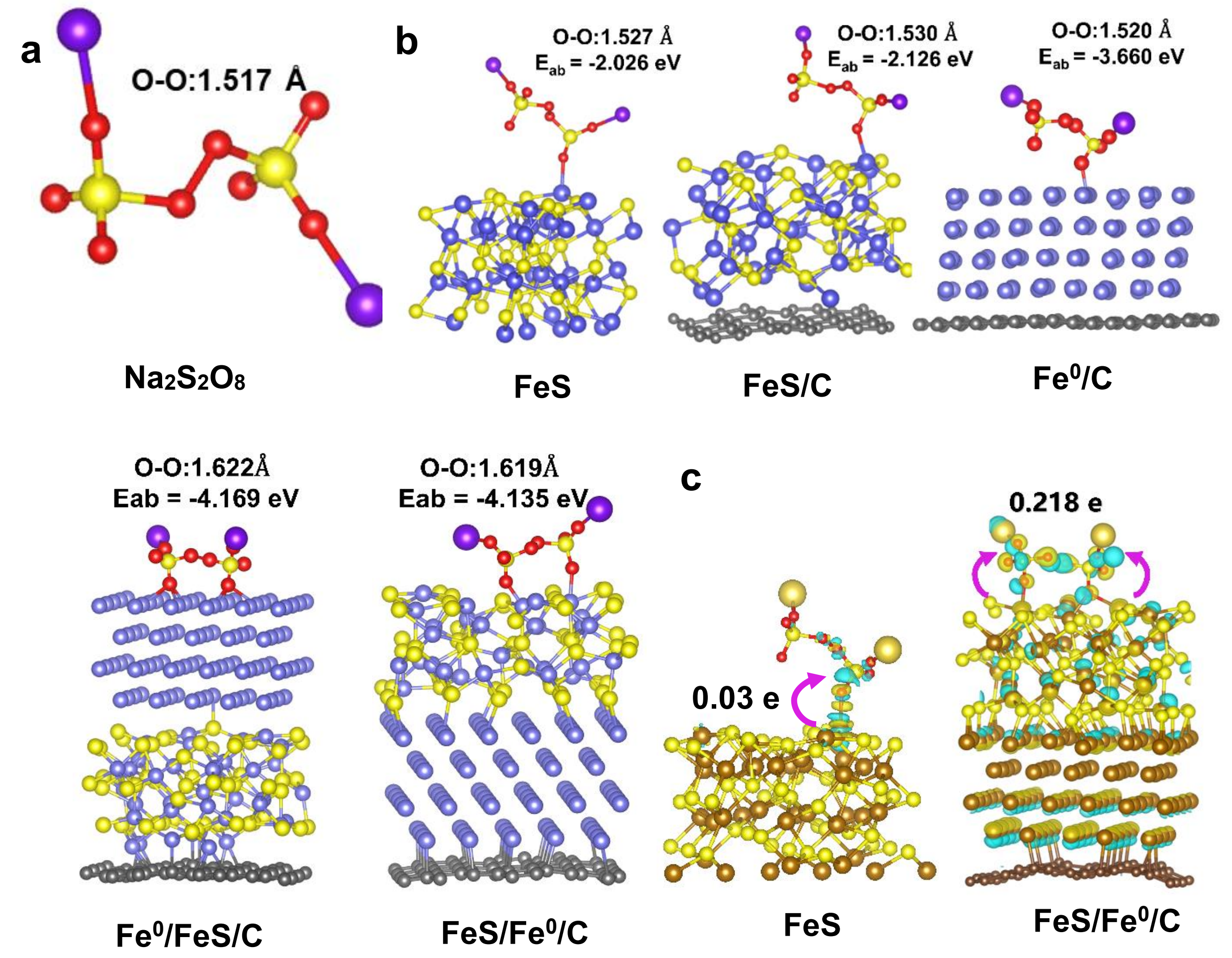

Supplementary Figure 30 l a The optimized adsorption structures of PDS. **b** The optimized adsorption structures system of different components with PDS, including FeS, FeS/C, Fe^0/C, Fe^0/FeS/C, and FeS/Fe^0/C. **c** The electron density difference of PDS adsorption on FeS and FeS/Fe^0/C.

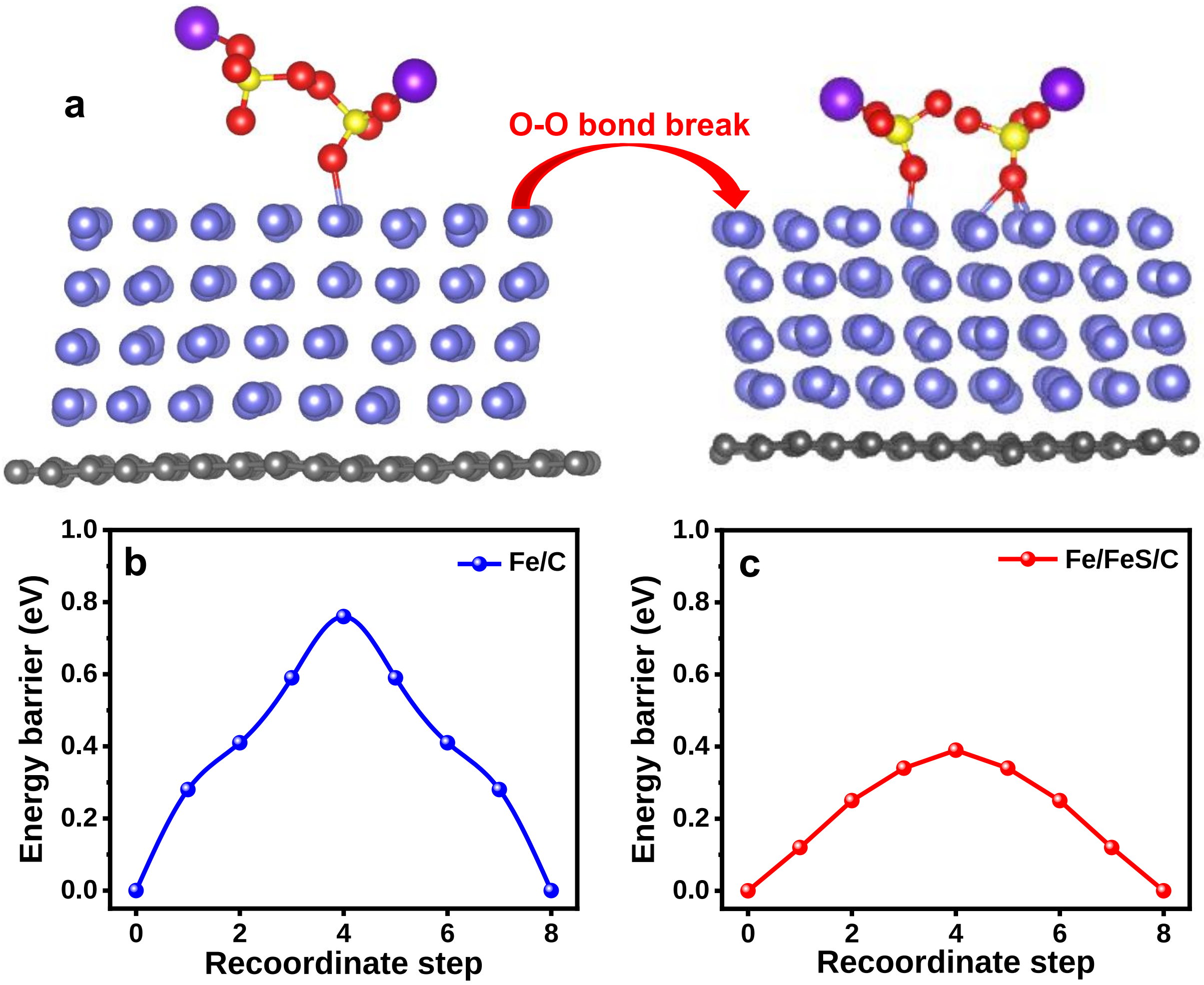

Supplementary Figure 31 I a The corresponding O-O bond breakage process of PDS for Fe/C. **b-c**, The energy barrier for the corresponding intermediate processes of O-O bond breakage of PDS on (**b**) Fe/C and (**c**) Fe^0/FeS/C.

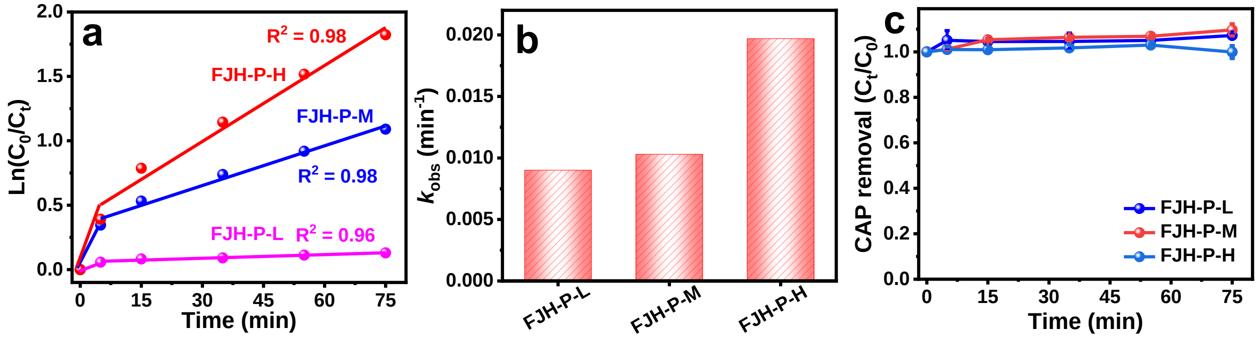

Supplementary Figure 32 I Kinetic analysis of CAP degradation and adsorption by the Fe-based material prepared at different FJH power. **a-b**, (**a**) $\ln(C_0/C_t)$ of CAP versus time and (**b**) k_{obs} of CAP degradation of Fe-based material prepared by different FJH power. **c** The CAP adsorption of Fe-based material prepared at different FJH power. The experiments were repeated twice. Degradation condition: $[CAP]_0$ = 60 mg L^{-1}, [material] = 1000 mg L^{-1}, [PDS] = 7 mmol L^{-1} (if need), initial pH_0 = 3.0±0.2, temperature = 28 ^oC.

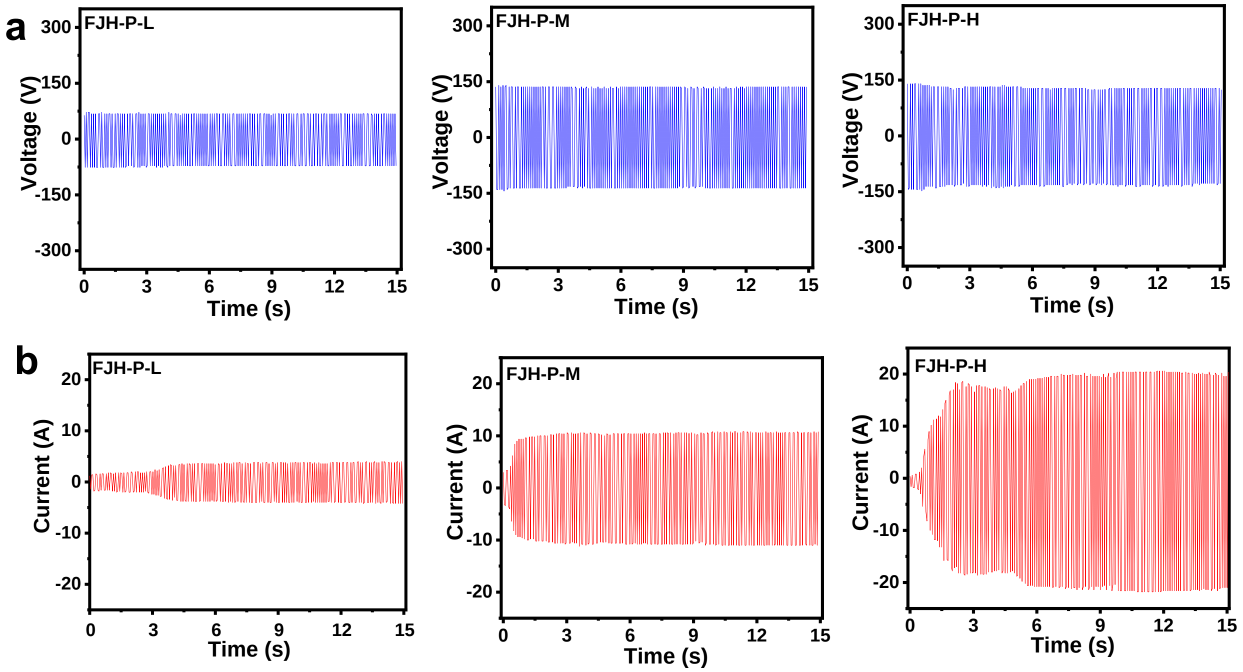

Supplementary Figure 33 I a-b, (**a**) Voltage and (**b**) current were recorded during the FJH process.

Supplementary Table 7 I The graphitization degree of FJH-P-L, FJH-P-M, and FJH-P-H by Raman measurements.

Samples	I_G/I_D	I_{2D}/I_G
FJH-P-L	0.47	-
FJH-P-M	0.90	0.50
FJH-P-H	1.21	1.43

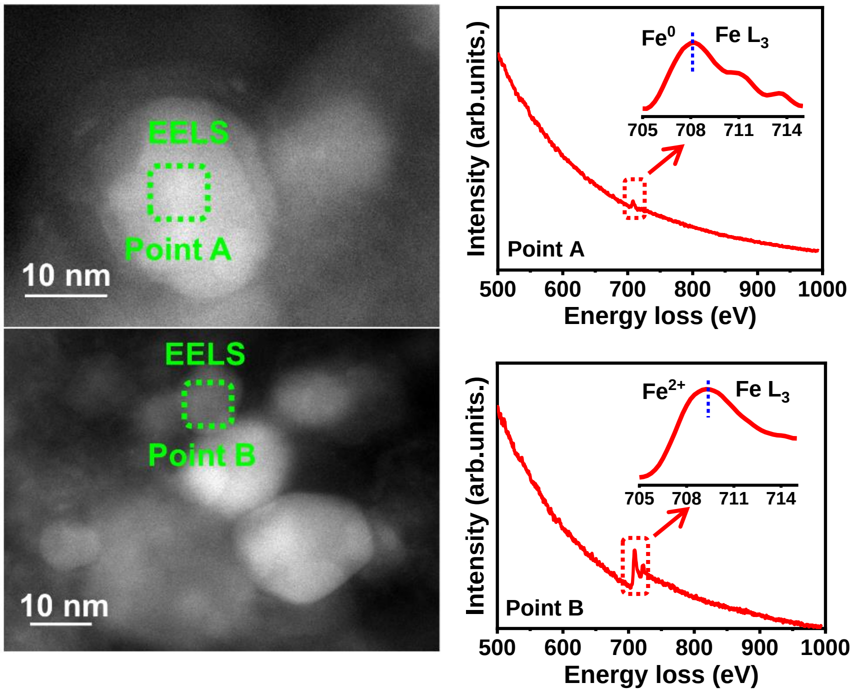

Supplementary Figure 34 I. Fe L_3 STEM-EELS data of FJH-P-H from point A and point B.

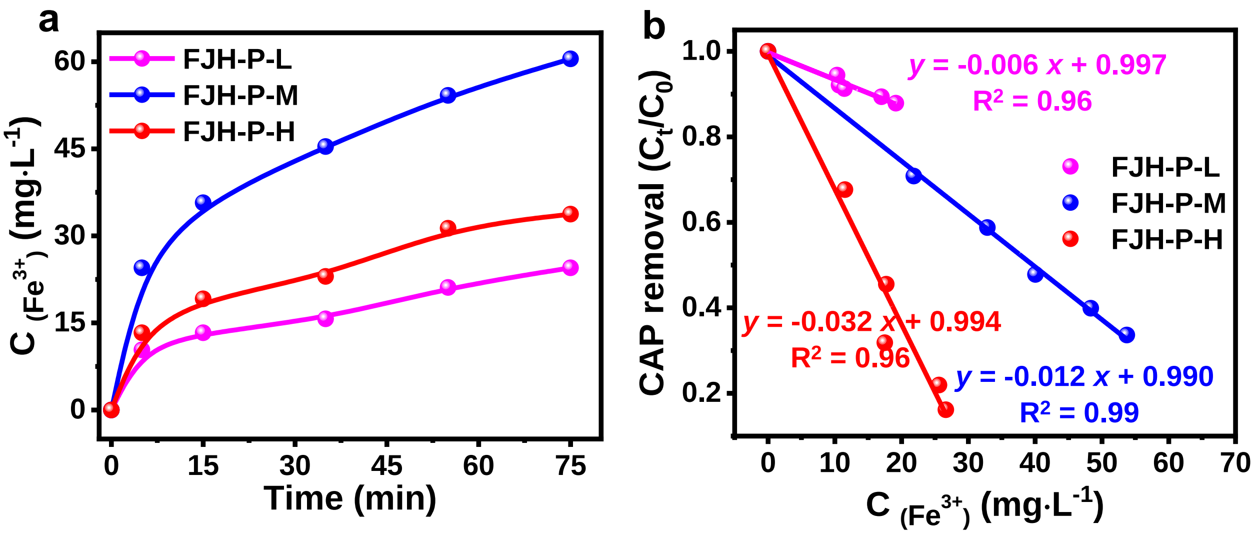

Supplementary Figure 35 I Transformation of Fe species in the degradation process. **a** Dissolving Fe^{3+} concentrations during the degradation process. **b** The linear relationships between Fe^{3+} concentration and degradation efficiency of CAP.

Supplementary Table 8 I The properties of different types of soil.

Soil	Fe content (wt %)	CEC cmol (+) kg^{-1}	Organic matter content (g Kg^{-1})	pH
Red soil	8.42	18.8	10.9	6.4
Yellow soil	2.78	19.3	39.2	5.6
Black soil	3.45	23.2	97.6	7.4

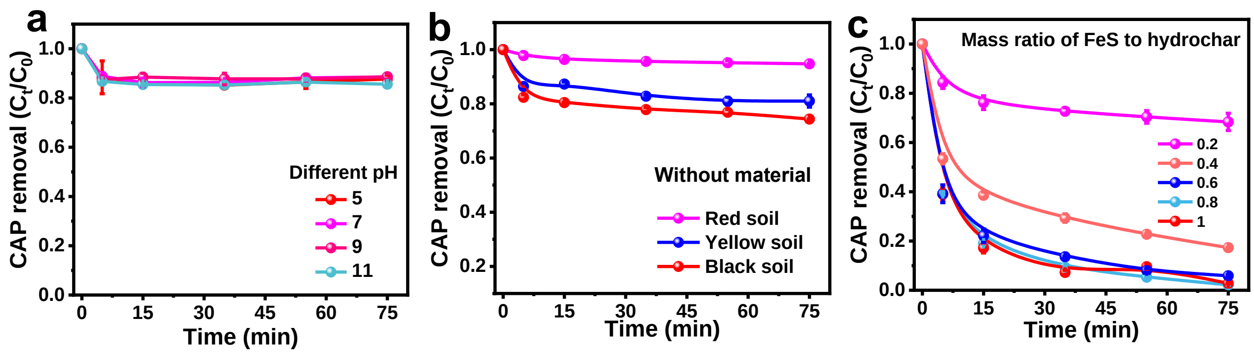

Supplementary Figure 36 I a The CAP adsorption of Fe-C-FJH at different pH. $[CAP]_0$ = 60 mg L^{-1}, [material] = 1000 mg L^{-1}, temperature = 28 ^oC. **b** The soil remediation application without a Fe-based material. Degradation condition: $[CAP]_0$ = 60 mg L^{-1}, [soil] = 100 g L^{-1}, [PDS] = 7 mmol L^{-1}. **c** CAP removal in aqueous solutions of Fe-based material prepared at different mass ratios of FeS/hydrochar by FJH. $[CAP]_0$ = 60 mg L^{-1}, [material] = 1000 mg L^{-1}, [PDS] = 7 mmol L^{-1}, initial pH = 3.0±0.2, temperature = 28 ^oC. The experiments were repeated twice (a, b, and c).

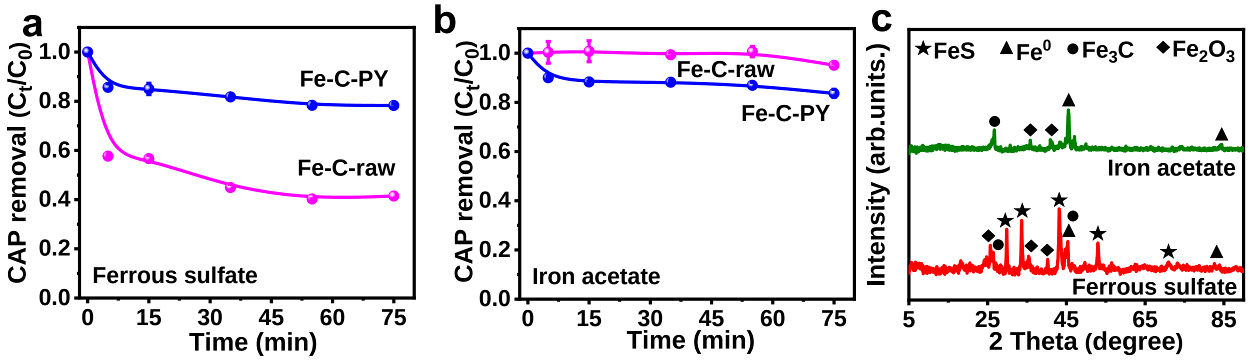

Supplementary Figure 37 I a-b, CAP removal of Fe-based material prepared by conventional pyrolysis with (**a**) ferrous sulfate and (**b**) iron acetate precursors. Degradation condition: $[CAP]_0$ = 60 mg L^{-1}, [material] = 1000 mg L^{-1}, [PDS] = 7 mmol L^{-1}, initial pH_0 = 3.0±0.2, temperature = 28 °C. The experiments were repeated twice (a and b). **c,** XRD analysis of Fe-based material produced from iron acetate and ferrous sulfate by FJH process. The prepared processes were the same as Fe-C-FJH.

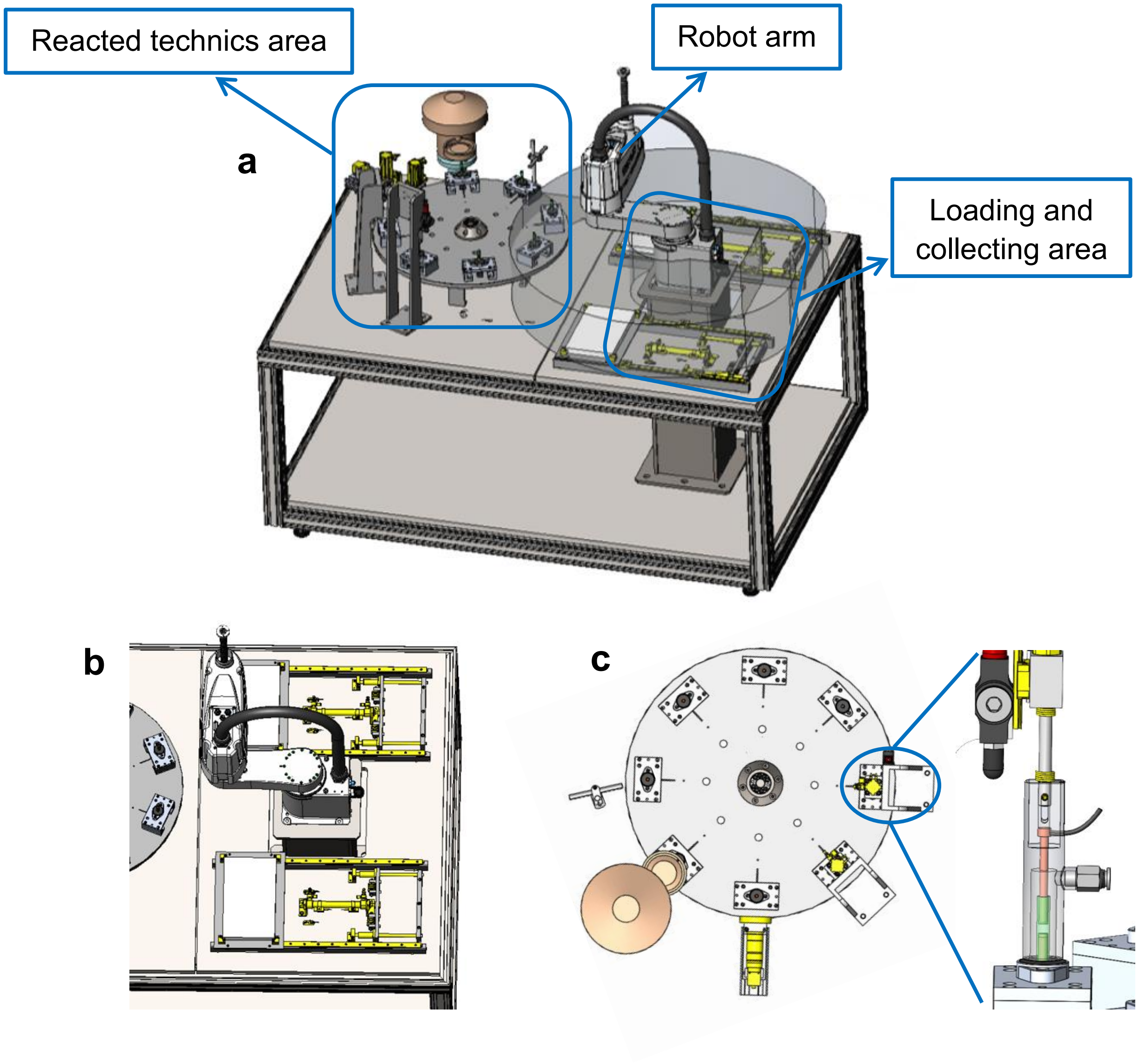

Supplementary Figure 38 I A large-scale production device of superior Fe-based material for industrialization by automation equipment. **a-c**, (**a**) The automation equipment, (**b**) the loading and collecting area with a robot arm, and (**c**) reacted technics area.

Supplementary Table 9 I The calculation of production efficiency by the automation equipment.

Loading mass (g)	Production yield (%)	Collecting mass (g)	Time (s)	Fe-based material (g day^{-1})
0.750	47.8	0.359	20	1549

Supplementary Table 10 I Fe-based material was prepared by different parameters of FJH.

Purpose	Fe-based material	Operation voltage (V)	External resistance (Ω)	Reaction time (s)
a	Fe-C-FJH	75	5	15
b	FJH-P-L	50	15	15
	FJH-P-M	100	10	15
	FJH-P-H	100	5	15

a. To investigate the advantages of FJH, Fe-C-FJH was prepared to compare with material by conventional pyrolysis.

b. To analyze the effects of FJH power on catalytic performances, Fe-based material was synthesized at different FJH power.

Supplementary References

1. Luong, D. X., *et al.* Gram-scale bottom-up flash graphene synthesis. *Nature*. **577**, 647-651 (2020).
2. Ravel, B. & Newville, M. ATHENA, ARTEMIS, HEPHAESTUS: data analysis for X-ray absorption spectroscopy using IFEFFIT. *J Synchrotron Radiat*. **12**, 537-541 (2005).
3. Zabinsky, S. I., Rehr, J. J., Ankudinov, A., Albers, R. C. & Eller, M. J. Multiple-scattering calculations of x-ray-absorption spectra. *Phys Rev B Condens Matter*. **52**, 2995-3009 (1995).
4. Shang, H., *et al.* Decarbonylation reaction of saturated and oxidized tar from pyrolysis of low aromaticity biomass boost reduction of hexavalent chromium. *Chem Eng J*. **360**, 1042-1050 (2018).
5. Yan, Q., *et al.* Constructing an acidic microenvironment by MoS_2 in heterogeneous Fenton reaction for pollutant control. *Angew Chem Int Ed Engl*. **60**, 17155-17163 (2021).
6. Wang, D., *et al.* Pyrogenic carbon initiated the generation of hydroxyl radicals from the oxidation of sulfide. *Environ Sci Technol*. **55**, 6001-6011 (2021).
7. Nie, M., *et al.* Degradation of chloramphenicol using a combination system of simulated solar light, Fe^{2+} and persulfate. *Chem Eng J*. **348**, 455-463 (2018).
8. Deng, B., Luong, D. X., Wang, Z., Kittrell, C., McHugh, E. A. & Tour, J. M. Urban mining by flash Joule heating. *Nat Commun*. **12**, 5794 (2021).
9. Kresse, G. & Joubert, D. From ultrasoft pseudopotentials to the projector augmented-wave method. *Phys Rev B*. **59**, 1758 (1999).
10. Kresse, G. & Furthmüller, J. Efficiency of ab-initio total energy calculations for metals and semiconductors using a plane-wave basis set. *Comp Mater Sci*. **6**, 15-50 (1996).
11. Kresse, G. & Furthmüller, J. Efficient iterative schemes for ab initio total-energy calculations using a plane-wave basis set. *Phys Rev B* **54**, 11169 (1996).
12. Perdew, J. P., Burke, K. & Ernzerhof, M. Generalized gradient approximation made simple. *Phys Rev Lett*. **77**, 3865 (1996).
13. Lee, K., Murray, É. D., Kong, L. Z., Bengt, I. L. & Langreth, D. C. Higher-accuracy van der Waals density functional. *Phys Rev B* **82**, 081101 (2010).
14. Tan, C., Dong, Y., Fu, D., Gao, N., Ma, J. & Liu, X. Chloramphenicol removal by zero valent iron activated peroxymonosulfate system: Kinetics and mechanism of radical generation. *Chem Eng J*. **334**, 1006-1015 (2018).
15. He, F., Ma, W., Zhong, D. & Yuan, Y. Degradation of chloramphenicol by alpha-FeOOH-activated two different double-oxidant systems with hydroxylamine assistance. *Chemosphere*. **250**, 126150 (2020).
16. Zhang, T., *et al.* Synergistic degradation of chloramphenicol by ultrasound-enhanced nanoscale zero-valent iron/persulfate treatment. *Sep and Purif Technol*.

240, 116575 (2020).

17. Liu, C., Wang, P., Qiao, Y. & Zhou, G. Self-assembled Bi_2SeO_5/rGO/MIL-88A Z-scheme heterojunction boosting carrier separation for simultaneous removal of Cr (VI) and chloramphenicol. *Chem Eng J*. **431**, 133289 (2022).
